# Coaching for a Sustainability Transition: Empowering Student-Led Sustainability Initiatives by Developing Skills, Group Identification, and Efficacy Beliefs

**DOI:** 10.3389/fpsyg.2021.623972

**Published:** 2021-05-05

**Authors:** Karen R. S. Hamann, Jana R. Holz, Gerhard Reese

**Affiliations:** ^1^Environmental Psychology Unit, Department of Social, Environmental, and Economic Psychology, Faculty of Psychology, University of Koblenz-Landau, Landau, Germany; ^2^Junior Research Group “Mentalities in Flux”, Institute of Sociology, Friedrich-Schiller-University Jena, Jena, Germany

**Keywords:** efficacy beliefs, sustainability volunteering, sustainability behavior, group identification, coaching program, university, student initiatives, multilevel perspective

## Abstract

Self-, collective, and participative efficacy are strong predictors of sustainability action. Yet, few studies have investigated the dynamics and variability of efficacy beliefs. In this transdisciplinary study, we tested such factors in the context of a peer-to-peer coaching program for sustainability volunteers, embedded in a structured-educational context. Over weekends, 2 qualified coaches trained 36 German bottom-up, student-led sustainability initiatives. These coaches instructed students in team building, envisioning, project planning, and on-campus sustainability practice. While 317 participants completed our pre-questionnaire, *N* = 165 completed both the pre- and post-questionnaire. As hypothesized, after having participated in the coaching weekend, action skills, collaboration skills, group identification, and self-, collective, and participative efficacy all increased. The latter of these increased, to our knowledge, for the first time in environmental psychology research. Group identification and having a vision emerged as important efficacy predictors, and participative efficacy beliefs in turn predicted volunteering. Moreover, we took initial steps in investigating the interaction of psychological and structural factors from a multilevel perspective. Our analyses revealed that efficacy beliefs on the individual level were higher when the university had a green office and when the student initiative was at a small university. We conclude by proposing an empowerment model for sustainability volunteers and by discussing the practical implications of our findings.

## Introduction

“*We already have all the facts and solutions. All we have to do is to wake up and change.”* (Greta Thunberg, TED, 2018) (Thunberg, 2018).

Sustainability movements, such as Fridays for Future, are on the rise, with continuous protests in more than 100 countries (BBC, 2019, 2020). The spotlights of such bottom-up initiatives tend to be focused on those select individuals who initiate and inspire others to join movements, while overlooking underlying structures and supporting people (e.g., the Fridays for Future organizers, or groups already pioneering sustainable forms of living). Yet, they too form an integral part of sustainability transitions.

According to Geels and Schot (2007), such sustainability transitions require change on multiple levels: socio-technical landscapes (e.g., long-term value patterns or demographic trends), socio-technical regimes (e.g., infrastructures or lifestyles), and niches (e.g., small networks producing radical innovations). From this multilevel perspective, socio-ecological transitions take place if niche alternatives are tested by pioneers and prepared to be embedded in, or even replace, socio-technical regimes. As such, we set out to examine the case of German bottom-up, student-led sustainability initiatives (niches) that were coached by *netzwerk n*, a non-profit organization that promotes sustainability throughout the education practices, operations, research, and governance of higher education institutions (university regime) (netzwerk n e.V., n.d.). In this transdisciplinary research project, we developed research questions and design conjointly with *netzwerk n* (see Lang et al., 2012). Results were later discussed at application-oriented conferences, and with members of *netzwerk n*, thus contributing to advancing the coaching program and transforming practices in sustainability initiatives in the future. From a scientific point of view, we were especially interested in explaining sustainability behavior at the individual and group level from a psychological perspective.

Specifically, we draw on recent models of collective action to examine the roles of self-, collective, and participative efficacy beliefs in the process of psychological and actual empowerment. To this end, we raise three main questions: Does a coaching program have the potential to empower its participants? Do group identification, collaboration skills, action skills, and envisioning a sustainable future enhance perceived efficacy? Do efficacy beliefs play a relevant part in activist motivation and activity? Throughout our study, we use Geels and Schot's (2007) multilevel perspective as a base for embedding our psychological perspective into the broader context of the socio-technical regimes and landscapes of universities. As most sustainability initiatives in our sample focus on the environmental dimension of sustainability, our literature review summarizes research on environmental activist, and pro-environmental behaviors.

### The Case of Environmental Activists

Representative studies suggest that the proportion of the German population volunteering for environmental and nature protection increased from 4 to 6% between 2006 and 2008 to 8–9% between 2010 and 2016 [Bundesministerium für Umwelt, Naturschutz, und nukleare Sicherheit [BMU] and Umweltbundesamt [UBA], 2015, 2017]. In 2014, 48% of all non-activists reported they could imagine actively engaging in environmental protection in the future. This potential for sustainability activism requires an understanding of the conditions for effective, satisfactory, and long-lasting active engagement in niches—a behavior rarely studied in psychological research (see Curtin and McGarty, 2016). In line with Curtin and McGarty (2016), we define activists as “people who actively work for social or political causes and especially those who work to encourage other people to support those causes” (p. 228). Intentionally, this definition includes both protesters who might put pressure on the socio-technical landscape, but also volunteers who sustain the organizational structures of social and ecological niches. As reflected in our study, university students are often presented as and targeted to be pioneers in socio-ecological transitions (UNESCO Global Action Programme ESD, n.d.). A major theme and driving force in an environmental activist's life is the feeling of efficacy (Martinez and McMullin, 2004; Almers, 2013). By focusing on psychological efficacy beliefs in a broader context of agency and empowerment in regimes and landscapes, our study sheds light on why sustainability volunteers become active, how their active engagement might be boosted, and how they influence sustainability transitions.

### Efficacy Beliefs at the Individual and Group Levels and Somewhere in Between

According to Bandura's self-efficacy theory (1997), self-efficacy refers to the belief that one can successfully execute the behaviors required to produce certain desired outcomes. In this respect, self-efficacy beliefs comprise some amalgam of agent-action-aim relationships. While Bandura (1997) focuses on agent-action self-efficacy (one's perceived ability to perform a behavior), other environmental psychology studies focus on agent-aim self-efficacy (see Hamann and Reese, 2020). In psychology literature, agent-aim efficacy is often termed self-efficacy (Hanss and Böhm, 2010), perceived (consumer) effectiveness (Lee et al., 2014), and response efficacy (Doherty and Webler, 2016). In the context of this paper, agent-aim self-efficacy may be an initiative member's perceived ability to change sustainability policies at their university. However, some authors argue that sustainability challenges require collective solutions and, as such, social identity factors should be taken into account (see Fritsche et al., 2018).

Tajfel (1978, p. 63) defines social identity as a combination of a person's perceived group membership and the emotional significance of that membership. In other words, it is the capacity to define oneself in terms of “we” instead of “I” (Fritsche et al., 2018). Building on this understanding, collective efficacy refers to a social group, i.e., a group member's belief in the group's ability to produce desired outcomes (Bandura, 1997). In the context of this study, this could be an initiative member's belief that their group has the ability to persuade their university to offer more sustainability-related courses. Then again, as suggested by Olson's paradox (1968), too much collective efficacy could eventually lead to inaction as a single member's behavior might seem unnecessary for goal achievement (Olson, 1968). Accounting for this, Van Zomeren et al. (2013) introduced participative efficacy, which is the belief that a person can make a significant contribution to the achievement of a group goal^1^.

Finally, several qualitative and quantitative studies suggest that efficacy-related affective states such as feeling hopeful, euphoric, moved, or enthusiastic, are associated with sustainability actions (Drury and Reicher, 2005; Drury et al., 2005; Ojala, 2015; Feldman and Hart, 2016; Coelho et al., 2017; Hamann and Reese, 2020; Landmann and Rohmann, 2020; but see van Zomeren et al., 2019). While Bandura (1997) and Coelho et al. (2017) view affective states as antecedents of efficacy beliefs, Landmann and Rohmann (2020) conceptualize them as mediators between collective efficacy and collective action. We follow Drury and Reicher's (2005) notion that cognitive efficacy beliefs and efficacy affect (e.g., feeling hopeful, enthusiastic) jointly constitute empowerment, and explore the role of efficacy affect in the interplay of efficacy beliefs and sustainability behavior.

### How the Agent-Aim Aspect of Efficacy Beliefs Relate to Sustainability Behaviors

Perceived efficacy is needed to increase climate mitigation behaviors (e.g., Intergovernmental Panel on Climate Change [IPCC], 2019, p. 364) as well as other sustainability behaviors. Whereas agent-action efficacy beliefs were investigated in a meta-analysis that provided support for its importance in environmental actions (see Bamberg and Möser, 2007), agent-aim efficacy beliefs have not received as much attention in the research community. As such, we present a brief overview of correlational and intervention studies. Based on a categorization by Stern (2000) and Homburg and Stolberg (2006), we contrast previous findings with four subtypes of pro-environmental behavior that we think are also suitable for the broader sustainability domain: private behavior (e.g., recycling), indirect behavior (e.g., encouraging others), and activism that is further divided into protesting (e.g., joining protests) and volunteering (e.g., organizing sustainability events). The division within the activism subtype aligns with former studies that distinguished between organizing and participative action (Alisat and Riemer, 2015), campaign and protest action (Amna, 2012), and institutionalized and non-institutionalized action (Van Stekelenburg et al., 2016). Yet, some authors doubt if such a differentiation is sensible as both protesting and volunteering can be viewed as collective action (see Kende, 2016; Van Zomeren, 2016; Thomas et al., 2017; Sabherwal et al., 2021). In this study, we have the opportunity to test if such a distinction has incremental value.

#### Private Behavior

While many studies find agent-aim self-efficacy to be an important predictor of private behavior such as energy saving behavior or sustainable consumption (Roberts, 1996; Straughan and Roberts, 1999; Kim and Choi, 2005; Hanss and Böhm, 2010; Lee et al., 2014; Hunter and Röös, 2016; Loy et al., 2020), others do not (Homburg and Stolberg, 2006; Kim, 2011; Chen, 2015; Wang and Lin, 2017). Regarding efficacy aims, Hanss and Böhm (2010) found that an indirect self-efficacy aimed at encouraging others to promote sustainable development was a better predictor of private behavior than self-efficacy aimed generally at promoting sustainable development (see also Hanss et al., 2016). However, another study produced the opposite result (Hamann and Reese, 2020). Collective efficacy seems to be a relevant predictor for private behavior such as the intention to use an electric vehicle (Homburg and Stolberg, 2006; Rees and Bamberg, 2014; Chen, 2015; Barth et al., 2016; Carmi and Mostovoy, 2017). Therefore, Jugert et al. (2016) propose the existence of a mediation path between collective efficacy and private behavior via self-efficacy. This was supported in their own and others' correlational research (e.g., Reese and Junge, 2017).

#### Indirect Behavior

While, in one study, self-efficacy generally aimed at protecting the environment did not seem to predict indirect behavior (Geiger et al., 2017), in another study, indirect self-efficacy aimed at encouraging others was its most important predictor (Hamann and Reese, 2020). An earlier study by Homburg and Stolberg (2006) revealed that collective efficacy also relates to indirect behavior.

#### Protesting and Volunteering

Though environmental protesting and volunteering seem to be best predicted by collective efficacy (Rees and Bamberg, 2014; Thomas and Louis, 2014; Besta et al., 2017; Sabherwal et al., 2021; for a meta-analysis beyond the environmental context, see Van Zomeren et al., 2008), self-efficacy is also a fairly good predictor (Brunsting and Postmes, 2002; Lee et al., 2014; Doherty and Webler, 2016). Recent studies found results favoring participative efficacy over collective efficacy as a predictor of protesting and volunteering (e.g., participation in transition town meetings), especially for participants who identified strongly with the cause (Bamberg et al., 2015; van Zomeren et al., 2019; Hamann and Reese, 2020).

To summarize this correlational research, there are mixed results for all behavior subtypes with a tendency for self-efficacy predicting private behavior, collective efficacy predicting protesting, and participative efficacy predicting volunteering. It also appears that outcomes are usually psychological and self-reported rather than structural and observable. Empowerment theory (Zimmerman, 1990) complements self-efficacy theory (Bandura, 1997) as empowerment is defined as a participative process through which people achieve greater control, efficacy, and social justice (Rappaport, 1987). It therefore explicitly includes structural aspects (such as influences from regimes and landscapes) alongside psychological aspects. Based on Cattaneo et al. (2014), we aimed to enrich the psychological field by assessing observable and structural changes. We looked at social media events and posts as well as an institution's establishing of a green office (a sustainability office funded and approved by a university; Rootability, n.d.) as observable outcomes of perceived efficacy.

### Efficacy Predictors—Many Suggestions, Few Empirical Studies

Bandura (1997) proposed four main predictors of efficacy beliefs: mastery experiences, social modeling, verbal persuasion, and physiological/affective states. Although useful, there is no evidence that this list is conclusive, and we are unaware of any empirical tests within environmental studies (but for political activism, see Evripidou and Drury, 2013). Except for the single substantial meta-analysis that demonstrated an association between efficacy beliefs and group identification (Van Zomeren et al., 2008), what we are now summarizing are largely untested psychological determinants of efficacy beliefs that are relevant to the coaching program and university context.

#### Action Skills and Envisioning

Many researchers have pointed out the importance of perceived knowledge and action skills. Almers (2013) describes action competence as a composition of several types of knowledge: knowledge of (1) problem causes and consequences (2), envisioning solutions (3), how conditions can change, and (4) implementation (see also Geller, 1995; Cattaneo and Chapman, 2010; Riemer et al., 2016; Vestergren et al., 2016). In the same vein, the interactional components of psychological empowerment in empowerment theory (skill development, critical awareness, and understanding of causal mechanisms) might serve as efficacy predictors (see also Zimmerman, 1990, 1995). Almers (2013) found that perceived knowledge relates to skills and confidence amongst sustainability volunteers. However, in their interviews, Drury et al. (2005) found that protesters cited knowledge only once as an empowering factor. Like Almers (2013), Drury and Reicher (2009) consider creating a vision of a better world a crucial efficacy predictor. Envisioning might be particularly facilitated if confronted with inspiring personalities like Greta Thunberg (Sabherwal et al., 2021). Developing a vision of sustainability solutions is also a critical function of niches in the multilevel perspective (Geels, 2011).

#### Group Identification

In literature on collective action, efficacy is oftentimes associated with and predicted by group identification (Drury and Reicher, 2005, 2009; Van Zomeren et al., 2008; Blackwood and Louis, 2012; Greenaway et al., 2015; Vestergren et al., 2016). Likewise, social support was frequently mentioned in qualitative interviews as a prerequisite for collective efficacy (Drury and Reicher, 1999; see also Babcicky and Seebauer, 2020). Other authors have underlined the following group cohesion characteristics as possible efficacy predictors: appreciation and encouragement from others (Drury and Reicher, 1999; Almers, 2013), reciprocity (Lubell et al., 2007; Collins et al., 2014), trust (Collins et al., 2014), and social norms (Van Zomeren et al., 2004; Doherty and Webler, 2016; Wang and Lin, 2017).

#### Collaboration Skills

Finally, some researchers discussed and examined various signs of collaboration skills as efficacy predictors. These include resource mobilization (Zimmerman, 1995), goal setting (Cattaneo and Chapman, 2010), other members' perceived expertise (Marks et al., 2001), group consensus (Bongiorno et al., 2016), conflict management (Riger, 1984; Peterson and Zimmerman, 2004), role clarity (Chen and Bliese, 2002; Harp et al., 2017), and opportunity role structure (i.e., accessibility of positions) (Peterson and Zimmerman, 2004). From a procedural perspective, collective action itself can serve as efficacy predictors (Swim et al., 2019).

In summary, among the manifold psychological efficacy predictors, mostly group identification and norms are quantitatively tested in the field of environmental studies. Thus, our study pioneers the testing of several psychological and structural efficacy predictors that might be particularly relevant for sustainability volunteers.

### Interventions: Efficacy Beliefs Crushed and Uplifted

In laboratory studies, agent-aim efficacy beliefs were successfully manipulated by highlighting behaviors and their impacts (Van Zomeren et al., 2010; Feldman and Hart, 2016; Jugert et al., 2016), using an environmental (loss) story frame (Morton et al., 2011; Steinhorst et al., 2015), with a behavior task of medium (vs. low or high) difficulty (Reese and Junge, 2017), with messages about non-violent protests (Thomas and Louis, 2014), and with discussions (Thomas et al., 2015). Other manipulations, such as providing favorable feedback (Doran et al., 2017), showing an activist video (regarding general efficacy measures, Landmann and Rohmann, 2020), and presenting hopeful messages (van Zomeren et al., 2019), were unsuccessful in promoting efficacy beliefs. A large-scale, knowledge-based, 8-week field intervention by Hanss and Böhm (2013) also failed to raise efficacy beliefs. Other research was hindered by baseline differences or lacked a pre- and post-test control group design (Bongiorno et al., 2016; Riemer et al., 2016). Taken together, efficacy manipulations produced mixed results, and there is a clear lack of field studies.

## Research Design

In cooperating with the NGO, *netzwerk n*, we had the unique chance of investigating a peer-to-peer coaching program for student-led sustainability initiatives, using pre- and post-questionnaire. During the coaching weekend, *netzwerk n* coaches (typically previously-trained students from other universities) visited 36 bottom-up student initiatives and instructed students in team building, envisioning, project managing, and on-campus sustainability. We decided to implement a voluntary pre- and post-questionnaire with a 6-month follow-up for all participants. This allowed for a strong empirical test of efficacy beliefs, group identification, and sustainability behaviors in the field, while at the same time providing a practically relevant evaluation.

### Hypotheses

The coaching program included elements of previously successful interventions and proposed efficacy predictors (e.g., conveying sustainability knowledge, modeling best practices from other universities, and acquiring new project management skills). For an overview of coaching methods, see Supplementary Table A1. Our empirical field study tests several hypotheses derived from theory:

- **Pre-post comparison**. The following factors are stronger after the coaching weekend than before it: psychological factors such as action skills, having a vision, group identification, collaboration skills, efficacy affect, efficacy beliefs, sustainability behavior, and volunteer time (1a), and observable factors such as social media events and posts (1b).
- **Efficacy beliefs as outcomes**. Efficacy beliefs are positively predicted by action skills, having a vision, group identification, and collaboration skills as psychological factors (2a), the existence of a green office as a regime factor (2b), and a smaller university (fewer students) and smaller town as landscape factors (2c). We expected volunteers in a small environment (e.g., small university) would be more likely to feel that their environment could be easily changed.
- **Efficacy beliefs as predictors**. Over and above other relevant covariates, efficacy beliefs positively predict sustainability behavior and volunteer time (3a), number of social media events and posts, and (3b) establishing of a green office (as indicator of a regime change) (3c). Compared to collective efficacy, self-efficacy is a stronger positive predictor of private behavior, and participative efficacy is a stronger positive predictor of volunteering (3d).

### Participants and Design

Throughout 2017 and 2018, student-led sustainability initiatives applied to participate in *netzwerk n* coaching programs. After admittance, *netzwerk n* would initiate a 2–4-day meeting between initiative members and two peer-to-peer coaches (students of another university). Four weeks before their coaching weekend, groups typically had a Skype meeting with their coaches and received an e-mail from the project coordinator with warm-up questions and an invitation to our pre-questionnaire (see Supplementary Material A2 for a description of the coaching weekend). Approximately one week after their coaching weekend, participants received an e-mail with information on next steps and our post-questionnaire.

Our final sample consisted of *N* = 341 members participating in *N* = 39 coaching weekends. Three groups took part in two coaching weekends. Of all participants, *N* = 317 completed our pre-questionnaire (196 females, 99 males; age *M* = 23.43 years, *SD* = 3.25), *N* = 193 finished our post-questionnaire (111 females, 52 males; age *M* = 23.28 years, *SD* = 3.03) on average 2 weeks after the coaching weekend, and *N* = 34 participated in the 6-month follow-up (22 females, 10 males; age *M* = 23.82 years, *SD* = 3.14). The large dropout rates are probably due to the voluntary nature of participation. On average, participants volunteered 5 h per week for their initiative during the pre-questionnaire, and 10% were paid to do so as part of a student job. The English version of the questionnaire was completed by 18 participants, and 19 initiative members had taken part in this particular coaching program before. After their coaching weekend, *N* = 30 coaching teams completed questionnaires regarding the coaching methods they employed.

### Measures

Given the transdisciplinary nature of this process, both our own scientific demands and the practical demands of *netzwerk n* were taken into account. All items were measured on 7-point Likert scales from 1 (*totally disagree/incorrect*) to 7 (*totally agree/correct*). Both pre- and post-questionnaires contained the following scales in the displayed sequence. Scale reliability was based on pre-questionnaire data. All item-scale correlations were larger than 30. As APA guidelines were followed for the ethical conduct of research, the questionnaires included an informed consent form. See Supplementary Materials A25 and A26 for the full questionnaire.

#### Action Skills

We constructed six items for action skills with reference to proposed efficacy predictors (α = 0.78, see e.g., Geller, 1995; Almers, 2013). These items reflect the knowledge and skills typically addressed in the coaching weekend (e.g., familiarity with sustainability concepts, and project management). Sample item: “I am familiar with sustainability at my university (e.g., organizational structures, environmental management systems, etc.).” We decided to use the term action skills instead of action competence as our measure captures precise knowledge and skills rather than an educational ideal (see Mogensen and Schnack, 2010).

#### Group Identification, Collaboration Skills, and Having a Vision

Four items measured identification with one's own sustainability initiative (α = 0.78) based on Cameron (2004, e.g., “I feel like I belong to the initiative”). Together with *netzwerk n*, we generated a scale for perceived collaboration skills, which incorporated theoretical propositions but had a low Cronbach's Alpha value due to its large spectrum of contents (α = 0.56, see e.g., Marks et al., 2001; Collins et al., 2014). Sample item: “I am satisfied with the communication structures of our initiative.” We included having a vision as a one-item efficacy predictor (“I have a vision of how a sustainable university could look”).

#### Efficacy Affect

Efficacy affect was measured by the following three items adapted from Hamann and Reese (2020, α = 0.84): “In my work for the initiative, I feel… motivated/hopeful/enthusiastic” (see also Feldman and Hart, 2016). Note that these items were only included for 32 coaching sessions.

#### Efficacy Beliefs

We adapted 13 items on sustainable development efficacy beliefs to our context (α = 0.87), which were derived from Hanss and Böhm (2010) and Van Zomeren et al. (2013). Agent-aim self-efficacy was captured in five items (α = 0.79), of which, two measured general sustainable development self-efficacy (e.g., “I, through individual actions, can promote sustainable development”), two measured an indirect self-efficacy to encourage others (e.g., “My sustainable action will encourage others to do the same”), and one measured university-specific self-efficacy (“I, through individual actions, can promote sustainable university development”). We operationalized agent-aim collective efficacy with five items that exactly mirrored the self-efficacy items (α = 0.87, e.g., “Through joint actions, we as an initiative can promote sustainable development”). Three items measured agent-aim participative efficacy (α = 0.88), of which, two addressed general participative efficacy (e.g., “I, as an individual, can make a significant difference, so that we, as an initiative, can promote sustainable development”) and one addressed university-specific participative efficacy (“I, as an individual, can make a significant difference, so that we, as an initiative, can promote sustainable university development”).

#### Sustainability Behavior and Volunteer Time

Sustainability behavior was measured with nine items (α = 0.72). We captured private behavior in three consumption-related items that reflected the ecological dimension of sustainability and were adapted from Kaiser et al. (2010, e.g., “I mainly buy seasonal food,” α = 0.73). We operationalized indirect behavior according to Homburg and Stolberg (2006) with two items (e.g., “I try to convince my friends and family members of the importance of sustainable development,” *r* = 0.44). Protesting [e.g., “I participate in protests (demonstrations, rallies, occupations, etc.) that promote sustainable development,” *r* = 0.42] and volunteering (e.g., “I organize educational events about sustainability topics,” *r* = 0.38) were measured with two items each, taken from Alisat and Riemer (2015). Based on Mazzoni et al. (2015), we asked participants how many hours per week they were working or volunteering for their initiative.

#### Single Measures and Demographics

For exploratory purposes, we inquired about participants' environmental identity (“I think of myself as an environmentally-friendly person,” see Lauren et al., 2016), stakeholder efficacy (“I feel able to contact stakeholders of my university”), volunteer burnout (“I feel burned out because of my commitment,” see Skaalvik and Skaalvik, 2007), volunteer payment, and demographics (age, gender, semester). For our collaboration partner, *netzwerk n*, questionnaires also contained evaluation items, e.g., “Coaches met our group needs.”

#### Coach Questionnaire

Coaches received questionnaires asking them to indicate which of the listed standard *netzwerk n* coaching methods they employed (yes/no) (see Supplementary Table A1). In addition, coaches were asked if they themselves were satisfied with their coaching weekend.

#### Social Media and Structural Variables

In order to assess changes in student initiative niches, we collected data on how many Facebook events (excluding internal group meetings) and posts (excluding re-posts) the student initiatives generated in the 1.5 years following (*N* = 35) and preceding the coaching (*N* = 30, initiatives were excluded if they had not owned an account for the 1.5 years preceding the coaching). For exploratory purposes, we further divided events into educational events (e.g., climate lectures), action events (e.g., upcycling workshops), university discussion events (e.g., discussions with other status groups), and protest events (e.g., preparing for Fridays for Future).

In order to capture landscape and regime influences, we gathered information on each university's student population, city population, number of staff members, student-staff ratio, number of professors, student-professor ratio, budget, budget-student ratio, the year in which the university was founded, and the gender of the university's president (based on most recent information). Moreover, we included whether the institution was a university or university of applied sciences, state or privately funded, located in former Western or Eastern Germany, and focused more on humanities or natural sciences. For depicting structural changes, we further coded if there had been a green office before and/or after the coaching, which was part of the institution (e.g., with permanent employees). Data was collected in 2020, dependent on online availability, and was supplied by three coders. Each data point was coded by at least two coders and inconsistencies were resolved by personal exchange.

### Data Analysis

**Figure 1:**
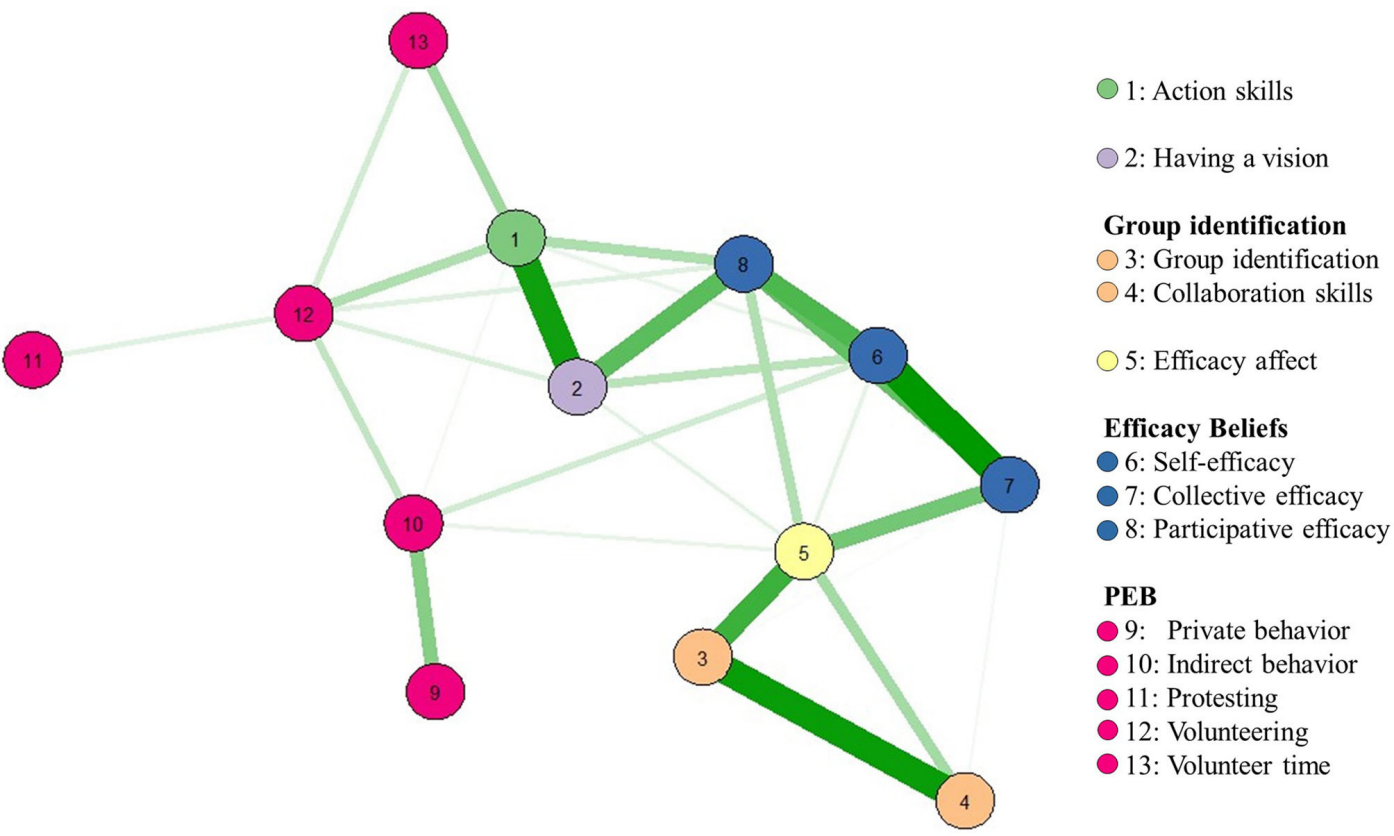
Visualization of bivariate square-transformed scale relationships (*r* > 0.30) with Gaussian Graphs as proposed by Bhushan et al. (2019). One-item measures have been inverted.

We performed data analysis with R Statistics version 3.6.0, and we performed data management with SPSS 25. We provide a trimmed dataset, script, and further analyses on OSF (see reference section, Hamann et al., 2019). For psychological hypotheses, we used multilevel modeling and report pseudo *R*^2^ according to Raudenbush and Bryk (2002), which is a measure indicating the proportion of the variance that predictor variables explain in the outcome variable. Furthermore, we examined H1 with *t-*tests and H2/3 with latent change models. For multilevel models of H1, and latent change models, we used our pre-post sample with *N* = 165, in which 12 participants were excluded beforehand because the time lag exceeded 4 months. H2/3 were tested with *N* = 310 pre-questionnaire participants (7 were excluded because they did not report their university). We detected no multivariate outliers using Mahalanobis distance when overall scales were included. When subscales were used for Mahalanobis distance, it was suggested that five participants be excluded. We checked main analyses without these outliers, but no differences occurred. Because of skewed distributions, we repeated every analysis with square-transformed scales. Unless otherwise noted, square-transformed scales produced similar results. To control for error accumulation in our hypotheses, we suggest a Bonferroni correction that divides *p* by the number of hypotheses. Therefore, a significant relation is signaled by *p* < 0.025 for 2 hypotheses in H1b, H2c, H3a/b/d, by *p* < 0.0125 for 4 hypotheses in H2a, and by *p* < 0.006 for 8 hypotheses in H1a. Figure 1 shows correlations of our main constructs. Means, standard deviations, and correlations of scales can be found in Supplementary Tables A3, A4 and Supplementary Figure A5.

## Results

### Confirmatory Factor Analysis

We first confirmed the proposed factor structure of efficacy beliefs and sustainability behavior. Regarding efficacy beliefs, a 5-factor model with self-, collective, and participative efficacy as latent factors and 2 nested factors (1 for 3 university-specific items and 1 for 4 efficacy items with the goal to encourage others; CFI = 0.983, RMSEA = 0.048, AIC = 11,517) fit the data better than a 3-factor model with only self-, collective, and participative efficacy (CFI = 0.913, RMSEA = 0.099, AIC = 11,666) and a 1-factor solution (CFI = 0.706, RMSEA = 0.178, AIC = 12,126). For sustainability behavior, the best fitting model was a 4-factor solution with a private, indirect, protesting, and volunteering factor (CFI = 0.912, RMSEA = 0.088, AIC = 9,874). This model fit the data better than both a 3-factor solution with a private, indirect, and protesting/volunteering factor (CFI = 0.857, RMSEA = 0.105, AIC = 9,903) and a 1-factor solution (CFI = 0.620, RMSEA = 0.162, AIC = 10,034). CFAs are portrayed in Supplementary Figures A6 and A7.

### Multilevel Analyses

Including a subject level and a sustainability coaching (group) level, we first calculated intraclass correlation coefficients (ICCs) for our main constructs, efficacy and sustainability behavior. Concerning pre-post comparisons, the subject level explained 32% of the variance in efficacy beliefs and 53% in sustainability behavior, while the group level explained 14% of variance in efficacy beliefs and 16% in sustainability behavior. Looking at all pre-participants, the group level explained 3% of the variance in efficacy beliefs and 12% in sustainability behavior. We included both levels in our analyses. For H2 and H3, we created predictors centered at the group-mean (see Tabachnick and Fidell, 2013). For H1, and partially H2b with dichotomous predictors, level 1 residuals were set to 0, and pseudo *R*^2^ could not be calculated.

### Hypothesis 1: Pre-post Comparison

**Figure 2:**
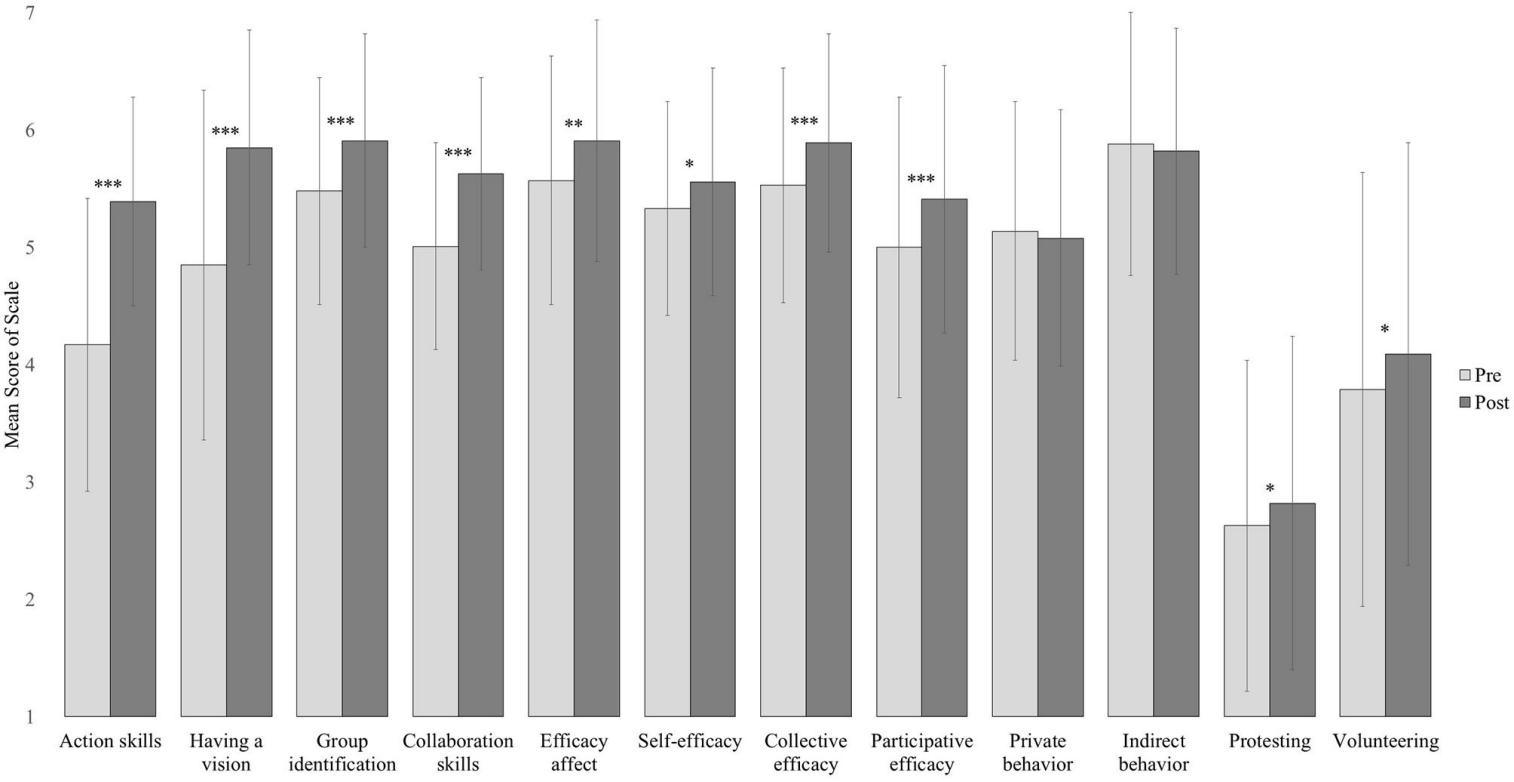
Average pre- and post-questionnaire scores of participants that took part in both (*N* = 165). Error bars indicate standard deviations. *p < 0.05, **p < 0.01, ***p < 0.001 (before Bonferroni correction).

Multilevel models revealed that the following psychological constructs of H1a were significantly higher after the coaching weekend than before it (see also Figure 2): action skills (*b* = 1.14 [0.95, 1.34], *t*(162) = 11.62, *p* < 0.001), having a vision (*b* = 0.96 [0.71, 1.21], *t*(162) = 7.48, *p* < 0.001), group identification (*b* = 0.38 [0.19, 0.58], *t*(163) = 3.91, *p* < 0.001), collaboration skills (*b* = 0.63 [0.46, 0.80], *t*(162) = 7.38, *p* < 0.001), efficacy affect (*b* = 0.30 [0.10, 0.51], *t*(133) = 2.93, *p* = 0.004), and efficacy beliefs (*b* = 0.28 [0.14, 0.42], *t*(162) = 3.95, *p* < 0.001). Yet, the change in volunteer time did not pass Bonferroni correction (*M*(*SD*)_pre_ = 4.88 (3.36), *M*(*SD*)_post_ = 5.34 (3.69), *b* = 0.50 [0.08, 0.92], *t*(161) = 2.35, *p* = 0.020), and sustainability behavior did not change significantly (*b* = 0.06 [−0.06, 0.18], *t*(150) = 1.05, *p* = 0.298). *Post-hoc* analyses demonstrated that self-efficacy (*b* = 0.18 [0.03, 0.18], *t*(162) = 2.34, *p* = 0.020), collective efficacy (*b* = 0.33 [0.16, 0.49], *t*(162) = 3.88, *p* < 0.001), participative efficacy (*b* = 0.38 [0.20, 0.57], *t*(162) = 4.06, *p* < 0.001), protesting (*b* = 0.21 [0.03, 0.38], *t*(161) = 2.31, *p* = 0.022), and volunteering (*b* = 0.30 [0.07, 0.53], *t*(161) = 2.55, *p* = 0.012) all increased. No changes emerged for private (*b* = −0.10 [−0.25, 0.05], *t*(150) = −1.29, *p* = 0.198) or indirect behavior (*b* = −0.10 [−0.26, 0.06], *t*(161) = −1.22, *p* = 0.224). We repeated and confirmed analyses with square-transformed scales and paired *t-*tests (see Supplementary Tables A8 and A9). Due to a low sample size in our follow-up questionnaire, we report long-term data for exploratory purposes only. Significant pre- vs. follow-up differences emerged for participatory efficacy and volunteering (*p* < 0.05) but appeared only descriptively in other constructs (see Supplementary Table A10 for more detailed descriptive analyses).

We tested H1b with dependent *t-*tests in a sample that consisted of *N* = 28 universities as subjects. Supporting our hypothesis, student initiatives hosted more Facebook events in the 1.5 years following coaching (*M*_post_ = 17.43, *SD*_post_ = 13.11) than in the 1.5 years preceding coaching (*M*_pre_ = 12.04, *SD*_pre_ = 8.07, *t*(27) = 2.31, *r* = 0.406, *p* = 0.029), yet this change did not pass Bonferroni correction. In *post-hoc* analyses, we found that, while there was an increase in the number of all event types, only the number of educational events increased significantly: educational events (*M*_pre_ = 6.64, *M*_post_ = 10.29, *r* = 0.399, *p* = 0.032), action events (*M*_pre_ = 4.68, *M*_post_ = 6.36), university discussion events (*M*_pre_ = 0.18, *M*_post_ = 0.36), and protest events (*M*_pre_ = 0.61, *M*_post_ = 0.79). For Facebook posts, there was no significant increase in the number of posts after coaching (*M*_post_ = 115.11, *SD*_post_ = 67.38) compared to before coaching (*M*_pre_ = 105.37, *SD*_pre_ = 85.95, *t*(26) = 0.63, *r* = 0.123, *p* = 0.534).

### Hypothesis 2: Efficacy Beliefs as Outcomes

Partially supporting H2a, having a vision and group identification significantly predicted efficacy, whereas collaboration skills did not, and action skills did not pass Bonferroni correction (overall pseudo *R*^2^ = 0.218, see Table 1). *Post-hoc* analyses revealed that this result was valid for both self- and participative efficacy (*p* < 0.05, see Supplementary Tables A11–13). For collective efficacy, group identification (*b* = 0.18 [0.05, 0.32], *t*(259) = 1.51, pseudo *R*^2^ = 0.023, *p* = 0.009) and collaboration skills (*b* = 0.19 [0.05, 0.33], *t*(258) = 2.58, pseudo *R*^2^ = 0.021, *p* = 0.001) were significant predictors, while action skills and having a vision were not (*p* < 0.05) (overall pseudo *R*^2^ = 0.139).

**Table 1:** Fixed effect predictors of efficacy beliefs for pre-questionnaire participants.

	***b***	**95% *CI***	***SE b***	***df***	**t**	**Pseudo *R*^**2**^**	***p***
(Intercept)	5.32	5.21, 5.42	0.05	31	103.48		<0.001
Action skills	0.10	0.01, 0.19	0.05	270	2.20	0.014	0.029
Having a vision	0.12	0.05, 0.19	0.04	270	3.33	0.036	0.001
Group identification	0.22	0.10, 0.34	0.06	270	3.67	0.044	<0.001
Collaboration skills	0.07	−0.05, 0.19	0.06	270	1.08	<0.001	0.282
Overall pseudo *R*^2^ = 0.218							

Confirming H2b, the existence of a green office prior to the coaching weekend positively predicted pre-questionnaire overall efficacy beliefs (*b* = 0.37 [0.07, 0.68], *t*(33) = 2.51, *p* = 0.017). Interestingly, the existence of a green office also predicted collective efficacy, participative efficacy, action skills, collaboration skills, and volunteer time (*p* < 0.05 for all). In line with H2c, a smaller student population at the university positively predicted higher pre-questionnaire efficacy beliefs (*b* = −0.01 [−0.02, −0.005], *t*(282) = −3.20, pseudo *R*^2^ = 0.012, *p* = 0.002). However, this was not the case for a smaller city population (*b* = −0.04 [−0.15, 0.07], *t*(33) = −0.78, pseudo *R*^2^ < 0.001, *p* = 0.439). Exploratory analyses showed that the effect of university size on efficacy beliefs was also reflected in associations of efficacy beliefs with the number of staff members, number of professors, and the budget (*p* < 0.05 for all), while other structural variables (e.g., year in which the university was founded) did not predict efficacy beliefs (*p* > 0.05 for all).

### Hypothesis 3: Efficacy Beliefs as Predictors

In accordance with H3a, efficacy beliefs significantly predicted overall sustainability behavior over and above other covariates (*b* = 0.17 [0.03, 0.32], *t*(233) = 2.30, pseudo *R*^2^ = 0.019, *p* = 0.022). Efficacy affect (*b* = 0.18 [0.06, 0.30], *t*(231) = 2.97, pseudo *R*^2^ = 0.033, *p* = 0.003) and action skills (*b* = 0.24 [0.14, 0.34], *t*(231) = 4.58, pseudo *R*^2^ = 0.080, *p* < 0.001) emerged as additional significant predictors of sustainability behavior, whereas having a vision (*b* = 0.08 [−0.001, 0.16], *t*(231) = 1.91, pseudo *R*^2^ = 0.011, *p* = 0.057), group identification (*b* = 0.01 [−0.13, 0.15], *t*(231) = 0.11, pseudo *R*^2^ = 0.004, *p* = 0.910), and collaboration skills (*b* = −0.12 [−0.26, 0.02], *t*(231) = −1.72, pseudo *R*^2^ = 0.009, *p* = 0.086) did not (overall pseudo *R*^2^ = 0.288). For volunteer time, efficacy beliefs did not turn out to be a significant predictor (*b* = −0.25 [−0.84, 0.33], *t*(227) = −0.84, pseudo *R*^2^ = < 0.001, *p* = 0.403, see Supplementary Tables A14 and A15).

Since, for both H3b and H3c, our dependent variable was group-based, we aggregated our pre-questionnaire independent variables at the group level (as more initiative members participated in them), ran regression analyses with White's adjustment for heteroscedasticity, and report HC3 as recommended by Foster-Johnson and Kromrey (2018). Efficacy beliefs did not predict post coaching Facebook posts (*b* = 48.78 [−4.55, 102.11], *t*(31) = 1.87, *R*^2^ = 0.076, *p* = 0.072), Facebook events (*b* = 6.58 [−8.25, 21.41], *t*(32) = 0.90, *R*^2^ = 0.037, *p* = 0.373), or establishing of a green office (*b* = −0.45 [−2.53, 1.64], *z*(27) = −0.42, *p* = 0.676). Exploratory *post-hoc* analyses revealed that efficacy beliefs predicted post coaching action events, and that this effect was driven by self-efficacy and collective efficacy (*p* < 0.05 for all). Moreover, volunteer behavior predicted discussion events (*p* < 0.01), and volunteer time predicted Facebook posts (*p* < 0.05). Looking at descriptive results, establishing a green office was associated with lower pre-questionnaire self-efficacy (*M*_established_ = 5.07, *M*_not_established_ = 5.24), higher collective efficacy (*M*_established_ = 5.47, *M*_not_established_ = 5.31), lower participatory efficacy (*M*_established_ = 4.54, *M*_not_established_ = 4.88), and more volunteer time (*M*_established_ = 5.85, *M*_not_established_ = 4.13) that also emerged as marginally significant predictor (*p* = 0.063). Due to low and unbalanced group sample sizes, results should be interpreted with caution.

H3d tested if, compared to collective efficacy, self-efficacy was a better predictor of private behavior and participative efficacy was a better predictor of volunteering and volunteer time. As can be seen in Table 2, only efficacy affect but no subtype of efficacy beliefs predicted private behavior. In congruence with H3d, Tables 3, 4 show that participative efficacy turned out to be a main positive predictor of volunteering and volunteer time, together with action skills and group identification. Collective efficacy, self-efficacy, and collaboration skills partially emerged as negative predictors. *Post-hoc* analyses showed that self-efficacy, efficacy affect, and action skills predict indirect behavior (overall *R*^2^ = 0.18) and that participative efficacy and action skills predict protesting (overall *R*^2^ = 0.12, *p* < 0.008 for all predictors), see Supplementary Tables A16 and A17.

**Table 2:** Fixed effect predictors of private sustainability behavior (pre-questionnaire).

	***b***	**95% *CI***	***SE b***	***df***	***t***	**Pseudo *R*^**2**^**	***p***
(Intercept)	5.04	4.87, 5.21	0.09	30	58.96		<0.001
Self-efficacy	0.10	−0.10, 0.30	0.10	236	0.10	<0.001	0.320
Collective efficacy	0.14	−0.07, 0.36	0.11	237	1.27	0.003	0.204
Participative efficacy	−0.08	−0.23, 0.07	0.08	236	−1.00	<0.001	0.320
Efficacy affect	0.32	0.14, 0.50	0.09	236	3.46	0.045	<0.001
Action skills	0.09	−0.06, 0.24	0.08	236	1.14	0.001	0.256
Having a vision	0.05	−0.07, 0.17	0.06	236	0.78	<0.001	0.439
Group identification	−0.18	−0.39, 0.03	0.11	236	−1.65	0.007	0.100
Collaboration skills	0.01	−0.19, 0.22	0.11	237	0.14	<0.001	0.892
Overall pseudo *R*^2^ = 0.080							

**Table 3:** Fixed effect predictors of volunteering (pre-questionnaire).

	***b***	**95% *CI***	***SE b***	***df***	***t***	**Pseudo *R*^**2**^**	***p***
(Intercept)	3.66	3.32, 4.01	0.17	27	21.38		<0.001
Self-efficacy	0.11	−0.15, 0.38	0.14	228	0.84	<0.001	0.404
Collective efficacy	−0.38	−0.67, −0.09	0.15	228	−2.53	0.023	0.012
Participative efficacy	0.31	0.11, 0.51	0.10	227	2.96	0.033	0.003
Efficacy affect	0.18	−0.06, 0.42	0.12	228	1.44	0.005	0.150
Action skills	0.41	0.21, 0.61	0.10	227	3.92	0.059	<0.001
Having a vision	0.14	−0.02, 0.30	0.08	227	1.68	0.008	0.094
Group identification	0.37	0.09, 0.66	0.15	227	2.55	0.023	0.012
Collaboration skills	−0.42	−0.70, −0.14	0.15	228	−2.91	0.032	0.004
Overall pseudo *R*^2^ = 0.293							

**Table 4:** Fixed effect predictors of volunteer time (pre-questionnaire).

	***b***	**95% CI**	***SE b***	***df***	***t***	**Pseudo *R*^**2**^**	***p***
(Intercept)	4.77	4.12, 5.47	0.34	24	14.17		<0.001
Self-efficacy	−0.53	−1.04, −0.03	0.26	223	−2.03	0.015	0.043
Collective efficacy	−0.35	−0.90, 0.20	0.29	224	−1.22	0.003	0.225
Participative efficacy	0.64	0.26, 1.02	0.20	223	3.23	0.041	0.001
Efficacy affect	0.37	−0.08, 0.83	0.24	224	1.58	0.006	0.116
Action skills	0.78	0.39, 1.16	0.20	223	3.87	0.058	<0.001
Having a vision	−0.01	−0.32, 0.30	0.16	223	−0.06	<0.001	0.956
Group identification	0.59	0.05, 1.14	0.28	223	2.12	0.015	0.036
Collaboration skills	−0.83	−1.36, −0.30	0.28	224	−2.99	0.032	0.003
Overall pseudo *R*^2^ = 0.202							

### Latent Change Analyses of Hypotheses 2a and 3a

We used latent change modeling to examine relationships of changes in constructs and therefore divided action skills into sustainability knowledge and university-related skills. We used a random parceling approach and did not analyze university-related skills and volunteering as the assumption of a strong measurement variance was violated. In agreement with H2a, changes in efficacy beliefs were associated with changes in sustainability knowledge (self-efficacy: *r* = 0.75, *p* = 0.017; collective efficacy: *r* = 0.61, *p* = 0.020; participative efficacy: *r* = 0.75, *p* = 0.003) and group identification (self-efficacy: *r* = 0.69, *p* = 0.011; collective efficacy: *r* = 0.58, *p* = 0.016; participative efficacy: *r* = 0.70, *p* = 0.003). However, only a change in participative efficacy significantly correlated with a change in collaboration skills (*r* = 0.57, *p* = 0.017). Regarding H3a, a change in all efficacy beliefs and affect accompanied a change in private behavior (self-efficacy: *r* = 0.87, *p* = 0.027; collective efficacy: *r* = 0.706, *p* = 0.018; participative efficacy: *r* = 0.71, *p* = 0.019; efficacy affect: *r* = 0.67, *p* = 0.026) and indirect behavior (self-efficacy: *r* = 0.77, *p* = 0.024; collective efficacy: *r* = 0.59, *p* = 0.041; participative efficacy: *r* = 0.57, *p* = 0.039; efficacy affect: *r* = 0.69, *p* = 0.032).

### Further Exploratory Analyses

First, we checked if participants who filled out pre- and post-questionnaires differed from participants who only filled out pre-questionnaires. Indeed, the latter showed significantly lower sustainability behavior (*F*_(1,303)_ = 10.87, *p* = 0.001). This held true for all subscales (*p* > 0.05 for all). Then, we tested whether specific methods (e.g., envisioning, project management, and learning about university structures) had effects on respective psychological items (e.g., having a vision and perceived collaboration skills) and found that learning about best practices (*b* = 1.19 [0.53, 1.86], *t*(117) = 3.57, *p* < 0.001) and learning about university structures (*b* = 0.89 [0.24, 1.53], *t*(118) = 2.73, *p* = 0.007) showed the proposed method × pre-post interactions, as apparent in Supplementary Figures A18 and A19. Finally, we explored activist burnout and found that it was positively predicted by action skills and participative efficacy (*p* < 0.001 for both), whereas self-efficacy, collective efficacy, and collaboration skills seemed to buffer activist burnout as negative predictors (*p* < 0.05 for all, overall pseudo *R*^2^ = 0.191, see Supplementary Table A20 for effect sizes and Supplement A21 for further exploratory analyses).

## Discussion

Our field study tested whether it is possible to enhance sustainable development efficacy beliefs and sustainability behavior by means of a coaching program. We further examined how action skills, having a vision, group identification, and collaboration skills influence efficacy beliefs, and whether efficacy beliefs can explain sustainability behavior, social media activity, and structural changes.

### Summary of Main Results

In accordance with H1, action skills, having a vision, group identification, collaboration skills, efficacy affect, and efficacy beliefs (especially collective and participative efficacy) were significantly stronger after the coaching weekend than before it. Protesting, volunteering, and volunteer time descriptively increased, while no changes emerged for private or indirect behavior. Initiatives also generated more Facebook events and posts after coaching than before coaching. However, the effect remained insignificant, presumably due to low sample size. Consistent with H2, having a vision, group identification, the existence of a green office, and small university size positively predicted overall efficacy beliefs (and varied regarding efficacy subtypes). While city size did not relate to efficacy beliefs, the predictive value of action skills and collaboration skills differed in regression and latent change analyses. Regarding H3, efficacy beliefs predicted sustainability behavior, but not volunteer time, number of Facebook posts and events, or establishing a green office after coaching. Looking at specific efficacy types, self-efficacy only predicted private behavior in latent change, but not regression, models. For private and indirect behavior, efficacy affect seems to be more relevant. As expected, participative efficacy predicted volunteering and volunteer time. Intriguingly, collaboration skills, self-efficacy, and collective efficacy appeared to be negative predictors of volunteering and volunteer time. In the following discussion, we first focus on psychological processes and then integrate regime and landscape factors in our reasoning.

### A Coaching Program as a Means for Change

Extending findings from previous field studies (see Hanss and Böhm, 2013), the coaching program managed to increase efficacy beliefs and volunteering. To our knowledge, this is the first study to reveal increases of participative efficacy beliefs in the field of environmental studies. Private and indirect behavior were not affected. This might be due to the coaching program's primary focus being group processes and not individual and collective impacts, as was the case with successful laboratory studies (see Jugert et al., 2016). A group process focus might also explain why, compared to self-efficacy, collective and participative efficacy were more affected by the coaching program. Additionally, long-term analyses suggest that the time-span of 1–2 weeks between the coaching weekend and invitation to the post-questionnaire might have been too short to display changes in actual behaviors. Yet, there is evidence for long-term effects of participative efficacy and volunteering in our follow-up questionnaire (see Supplementary Table A10). We assume that, through the coaching program, groups gained skills to organize themselves in a sustainable, supportive, and productive way, which further promotes participative efficacy and volunteer commitment.

Typical for field studies, we cannot point directly to what caused these effects. However, as mentioned earlier, the coaching program had many characteristics of successful interventions and proposed efficacy predictors, such as group discussions (Thomas et al., 2015) or finding a common vision and goal (Drury and Reicher, 2009; Cattaneo and Chapman, 2010). Also, the peer-to-peer approach might have played a role. Fortunately, we were able to test the effects of the specific methods used in the coaching program. While learning about best practices and university structures led to perceived knowledge of them, all other methods revealed no significant interactions, and analyses hint at the possibility that coaches employed methods tailored to participants' pre-knowledge. It is the nature of this field setting that methods were not varied systematically and that they possibly interacted with one another as well as with the activities, setting, and coaches' personalities.

### Fostering Efficacy Beliefs, Sustainability Behavior, and Structural Changes

This field investigation contributes strongly to the study of efficacy predictors, thus expanding self-efficacy theory (Bandura, 1997) and empowerment theory (Zimmerman, 1990) in the field of environmental psychology. As proposed by various authors (Blackwood and Louis, 2012; Vestergren et al., 2016), our empirical analyses show that group identification relates to all types of efficacy. Individual action skills (especially sustainability knowledge) are important for self- and participative efficacy, while group-related collaboration skills are more relevant for collective efficacy. Latent change analyses support these findings and indicate that collaboration skills are also associated with participative efficacy. Moreover, having a vision appears to be an innovative and strong positive predictor of self- and participative efficacy, as is shown in recent work by Fernando et al. (2020). While action skills and collaboration skills are present in self-efficacy theory as mastery experience and verbal persuasion (Bandura, 1997) and in empowerment theory as perceived competence and skill development (Zimmerman, 1995), neither group identification nor having a vision plays a major role in either theory. These should receive more attention in future research practice.

In our study, efficacy beliefs show latent change but not multilevel-regression associations with private behavior. Then again, though volunteering was strongly predicted by participative efficacy in regression analyses, it was not testable in latent change analyses. These findings make it difficult to draw final conclusions. Efficacy affect strongly influences private and indirect behavior in both latent change and regression analyses, but any effect on volunteering or volunteer time, as proposed by Ojala (2015) and Drury et al. (2005), is canceled out by other variables, like participative efficacy (see van Zomeren et al., 2019). This is especially surprising given that efficacy affect was operationalized as feeling hopeful, motivated, and enthusiastic in connection to participants' volunteering. Exploratory analyses in Supplementary Table A22 suggest efficacy affect to be a strong possible predictor of all efficacy types (see also Bandura, 1997; Coelho et al., 2017), yet our study does not allow causal conclusions.

In line with former studies (Thomas and Louis, 2014; Besta et al., 2017), group identification relates positively to volunteering, volunteer time, and protesting, and yet, participative efficacy shows stronger relations, which suggests participative efficacy beliefs are directly related to collective action (see Van Zomeren et al., 2008). Likewise, action skills stand out as an important predictor of volunteering and protesting. We propose though that its strong predictive power is attributable to the opposite causal direction, “if I volunteer, I gain action skills.” This might, of course, also be true for efficacy beliefs (see Sitzmann and Yeo, 2013). Further, protesting and volunteering display the same predictor structure but yield separate factors. Thus, the question remains whether distinguishing between those two types of activism is useful in psychological research (see Thomas et al., 2017). Surprisingly, collective efficacy and collaboration skills turn into negative predictors of volunteering when tested simultaneously with participative efficacy and group identification, which lends support to Olson's paradox (Olson, 1968). If an initiative member perceives the group as very competent and effective, they may not feel the urge to act themselves.

From a multilevel perspective, an institution's green office functions as a structural catalyst. Besides predicting collective and participative efficacy beliefs, it is also associated with action skills, collaboration skills, and volunteer time. Looking at other regime and landscape variables, we are astonished that only university size and none of our other structural variables seem to play a crucial role in developing efficacy beliefs and other psychological motivators (e.g., year in which the university was founded, gender of the university's president, and university type). Regarding initiatives' media output, initiatives generated more Facebook posts and events (educational, actionable, discussion-based, and protesting) after coaching compared to before coaching, but efficacy beliefs only predict action events in *post-hoc* analyses. Explorative analyses reveal that more volunteering is associated with more discussion events, and that volunteer time relates to Facebook posts. The three types of efficacy beliefs show somewhat diverging relations to the establishment of a green office, yet volunteering time emerges as marginally significant predictor. Those findings suggest that more effort that is put into a sustainability initiative indeed has the potential to lead to media visibility and university regime changes. However, results should be interpreted with caution due to their exploratory nature and their low sample size in group-level analyses.

### A Multilevel Model of Empowerment in Sustainability Volunteers

**Figure 3:**
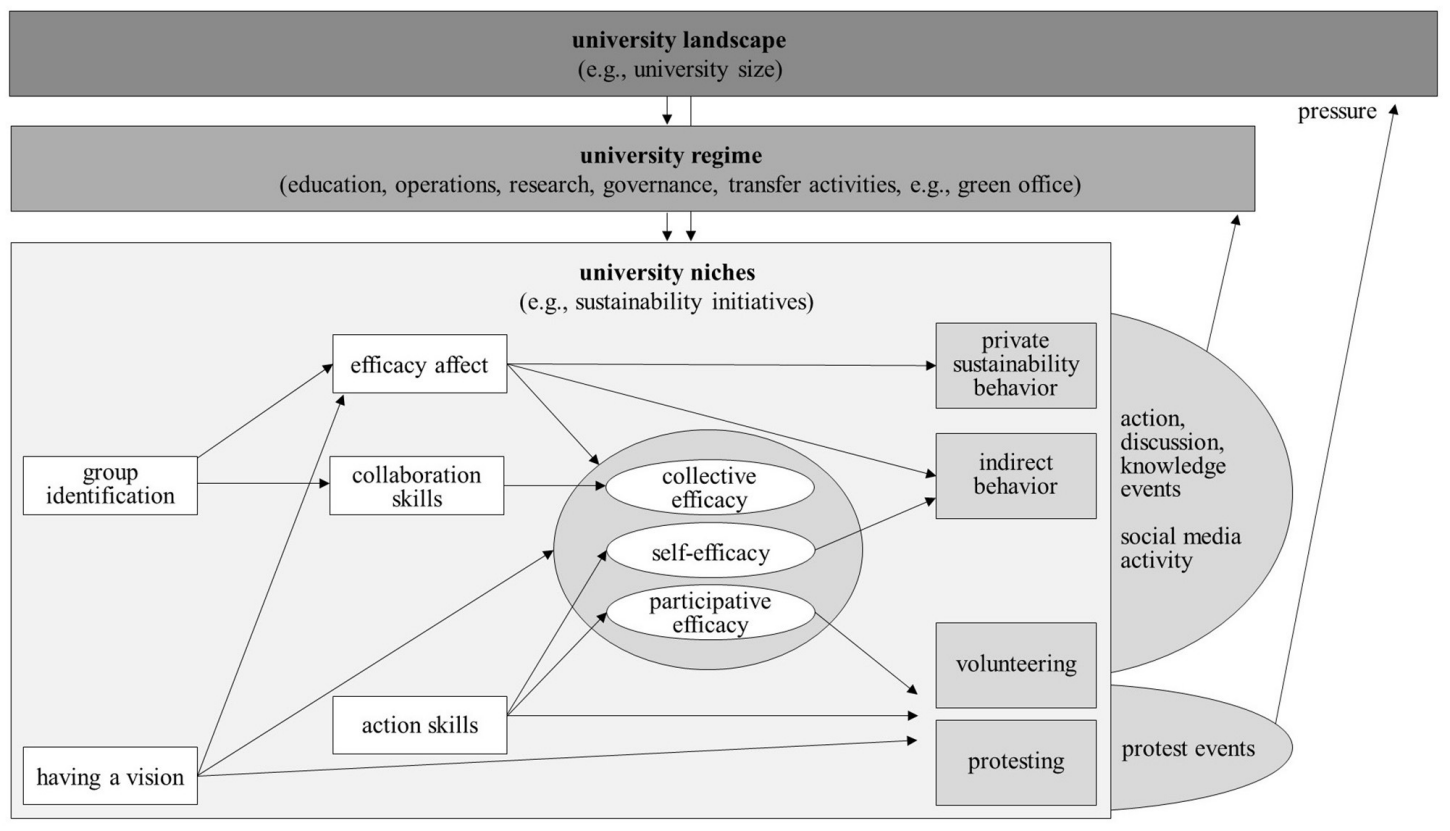
Multilevel model of empowerment in sustainability volunteers in a university context.

Our results emphasize that a distinction must be made between efficacy belief and sustainability behavior subtypes in order to determine the motivation of volunteers, and that structural regime and landscape factors are worth taking into account. Thus, we developed a theoretical and empirical model based on the social identity model of collective action [SIMCA] by Van Zomeren et al. (2008), the social identity model of pro-environmental action [SIMPEA] (Fritsche et al., 2018), and Geels' (2011) multilevel perspective (see Figure 3).

For sustainability volunteers as a niche group, efficacy beliefs (here, participative efficacy) predict collective action similar to SIMCA and SIMPEA. Group identification influences volunteering and protesting via efficacy affect and efficacy beliefs, suggesting that mediation paths for volunteers differ amongst newly founded groups, protesters, and laypeople [see EMSICA model by Thomas et al. (2012), Bongiorno et al. (2016), Landmann and Rohmann (2020), and Sabherwal et al. (2021)]. Action skills and having a vision emerge as predictors, both of which might be connected to appraisals and moralization (see Fritsche et al., 2018). Niche groups are influenced by university landscape and regime factors. They in turn influence regimes via events and social media activity and put pressure on the landscape by raising protest. As this model acknowledges intraindividual, interactional, behavioral, and structural correlates of efficacy beliefs, it is adaptable to empowerment theory (Zimmerman, 1990; Cattaneo and Chapman, 2010). Theoretical considerations and exploratory analyses leading to the psychological part of this model can be viewed in Supplementary Material A21.4.

Finally, as proposed by some authors, we explored efficacy beliefs as a buffer for activist burnout (Vestergren et al., 2016). Previous studies show a strong belief in one's own efficacy might serve as a buffer for burnout in teachers (Skaalvik and Skaalvik, 2007) and in older adults (Govindan, 1999). Our analyses show that action skills and participative efficacy are positively related to activist burnout. Probably, both lead to more volunteering behavior, which in turn prompts a physical and mental overload in the volunteers. However, both self- and collective efficacy, as well as perceived collaboration skills, were negatively associated with, and thus seem to buffer, activist burnout. This preliminary finding calls for extensive future research on the buffering function of specific efficacy types for activist burnout.

### Limitations and Future Directions

It is the nature of a field study that we cannot rule out alternative explanations. We would have liked to have included a (waiting list) control group in this study, but our specific sample of sustainability volunteers at universities prevented us from finding matching participants without having incentives to offer. Therefore, results must be interpreted with caution as we cannot draw causal conclusions or rule out that pre-post comparisons may be driven by exogenous variables (e.g., initiatives simply choosing to meet more frequently). Nevertheless, by analyzing coaching methods, using latent change analyses, and including structural variables, we gained knowledge of processes at work. Future research should examine coaching effects compared to a control group. A larger time-span between coaching weekends and post-questionnaires could uncover further changes in sustainability behavior. However, our follow-up dropout rate prevents a meaningful analysis in our case. A surprisingly large dropout occurred from pre- to post-questionnaire, and participants who dropped out before the post-test had significantly lower baseline rates regarding sustainability behavior. This might have influenced our results as it was probably the more engaged initiative members who participated in both questionnaires. We suggest that prospective studies provide an individual or group incentive for participation. Moreover, this study makes a first attempt to observe actual power regimes and shifts as a consequence of psychologically empowered people (see Cattaneo et al., 2014), however, the necessity of group-level analyses posed a threat to our results (see Foster-Johnson and Kromrey, 2018). Future studies should investigate larger group samples in order to understand the practical value of efficacy beliefs. In our study, a clear limitation is that some student initiatives only created one Facebook event for a “sustainability week” while others created events for each workshop within such a week. However, we think that this bias was mainly canceled out by the great number of events. Moreover, we only collected information on the quantity of posts, leaving out post quality. For future research, we think it would be promising to focus on efficacy affect as an efficacy belief predictor and mediator of efficacy-behavior relations, social norms (Doherty and Webler, 2016), and other group variables, like entitativity, permeability, and size (Lickel et al., 2000), and to explore more diverse efficacy goals since their predictive power seems to depend on the stage of commitment (Hornsey et al., 2006). Intrinsic motivation and need satisfaction could also be a worthwhile focus of future studies (Deci and Ryan, 2000; Boezeman and Ellemers, 2009), especially because our constructs already mirror elements of self-determination theory like the needs for competence (action skills, collaboration skills, efficacy beliefs), relatedness (group identification), and autonomy (efficacy beliefs).

## Conclusion and Practical Implications

This study tested the effects of a peer-to-peer coaching program on student-led sustainability initiatives. Contributing to self-efficacy theory in the field of environmental studies, it is the first field study to show changes in participative efficacy beliefs. Even if sample acquisition might be difficult, we encourage other researchers to investigate volunteers with practically important questions, like “How can people be motivated to volunteer in socio-ecological niches and keep up their (group) motivation?” Methods of the *netzwerk n* coaching program are available online, in German (netzwerk n e.V., 2018), and can be used for laboratory intervention testing as well as sustainability practice. Our results indicate that, in order to foster activism for sustainability, activists need to be psychologically and structurally empowered through a strong bond with their activist group, (learning) essential action skills, supportive institutions, like green offices, and circumstances that make them feel they can actually make a difference. With the below final quote, we would like to invite sustainability practitioners to pay special attention to group processes and embrace coaching opportunities.

*To realize in the here and now aspects of a world that does not yet exist (e.g., freedom, authenticity, equality) is to bring that world closer—through empowering its agents with the belief that they can create it. In a very concrete sense, then, social movement activists need to be architects of the imagination* (Drury and Reicher, 2009).

## Data Availability Statement

The datasets presented in this study can be found in an online repository at: https://osf.io/7k2zq/.

## Ethics Statement

Ethical review and approval was not required for the study setting in accordance with the local legislation and institutional requirements. The participants provided their written informed consent to participate in this study.

## Author Contributions

KH: study development, data collection, data analysis, and writing of manuscript. JH: study development, coordination of coaching program, data collection, and editing. GR: study development, editing, and supervision.

## Conflict of Interest

The authors declare that the research was conducted in the absence of any commercial or financial relationships that could be construed as a potential conflict of interest.

## Footnotes

^1^Within this study, self-, collective, and participative efficacy all pertain to agent-aim efficacy beliefs.
