# Supplementary material for: Coaching for a Sustainability Transition: Empowering Student-Led Sustainability Initiatives by Developing Skills, Group Identification, and Efficacy Beliefs
Source: Front Psychol. 2021 May 5;12:623972. doi: 10.3389/fpsyg.2021.623972 (PMC8131541; doi:10.3389/fpsyg.2021.623972)
Supplement: Supplementary file 1 [file Data_Sheet_1.pdf]

Coaching for a sustainability transition: empowering student-led sustainability initiatives by developing skills, group identification, and efficacy beliefs

Appendix/ Supplements

Table A1. Methods used in the coaching program and percentage of N=32 coaching week-ends in which they were applied. A precise description was published by network-n (2018).

name of method	label in dataset	description	% of N=32
Time-task matrix	Meth_ZeitAufMa	Tasks of group members are put into a timetable	94%
Sociometric lineup	Meth_SozMet	Group arranges itself on an imaginative line concerning sociometric questions	78%
Phrasing SMART goals	Meth_Smart	Group learns how to formulate SMART goals	75%
Competence figure	Meth_CompFig	Members draw a figure with their own competences and share it with the group	72%
Goal flower/ sun	Meth_Zielblume	Method for gathering and structuring group goals	69%
CANVAS method	Meth_Canvas	Method for brainstorming a specific project	63%
Stakeholder matrix	Meth_Stakeholder	Method for analyzing relevant stakeholders	59%
Learning about best practices	Meth_BestPrac	Input on best practices of other universities	50%
Pyramid technique for developing a vision	Meth_Pyra	Small groups brainstorm a vision, exchange and combine it with other groups	50%
Project-decision-matrix	Meth_ProEntMa	Method on prioritizing certain projects over other projects	47%
Imagination task	Meth_Traum	Members imagine the university of their dreams of the year 2030	44%
Project checklist	Meth_ProCheck	Method of structuring projects with bullet points	38%
Learning about sustainability	Meth_InputNA	Input on sustainable development	34%
Old stager, spring chicken	Meth_AlteHasen	Newer group members ask older group members questions	32%
Wander molecules	Meth_WaMo	Questions are asked in various group constellations	31%
Brainstorming on sus.	Meth_BrainNA	Brainstorming on sustainable development	22%
Exchange on project ideas	Meth_BestPro	Participants talk about project ideas and best practices	22%
Brainstorming on sus. university	Meth_BrainNAHoch	Brainstorming on sustainable university	19%

Learning about sus. university	Meth_InputNAHoch	Input on sustainable university	19%
Learning about uni structures	Meth_InputStruk	Input on university structures	19%
Journaling	Meth_Journaling	Group members answer some questions with creative writing	13%
Gallery walk	Meth_Gallery	A number of inputs are placed into a room, group members walk around and talk	13%
Exemplary sus. types	Meth_NATyp	Input on exemplary sustainability types	9%
Case study	Meth_CaseStudy	A case study is played as a game by group members	9%
Systemic lineup	Meth_SysAuf	Group arranges itself on an imaginative line concerning systemic questions	6%
World café	Meth_MdM	Group members present ideas and projects in an open format	3%
Stories of success and failures	Meth_Geschich	Stories of success and failures are shared in the group	3%
Future workshop	Meth_Zukunftswe	Method with a crisis, a envisioning, and a realistic phase	3%
Speakers corner	Meth_Speak	Members can pitch a project in a couple of minutes	3%
Energizer	Meth_Energizer	Also called warm-ups. Little games to make group members excited, calm, or concentrated.	<2: 9%<br>3: 37,5%<br>4: 37,5%<br>>5: 16%

Coaching example A2.
In order to provide a proper understanding of the coaching workshops, we describe one of them exemplarily:

On Friday afternoon, two trained coaches arrive at the university of Munich one hour before the coaching is about to start. The sustainability student initiative that applied for the coaching program has already organized a room – they managed to get into the student lounge instead of a usual seminar room! An initiative member who organized the coaching workshop opens the door and has a little chat with the peer coaches. While the two coaches prepare flipcharts and create a comfortable working atmosphere by setting up a circle of chairs with a little flower bouquet in the middle, little by little all eight initiative members arrive. Everyone settles in, grabs a cup of coffee or tea and seats themselves. The peer-to-peer coaching starts by letting everyone speak up once and tell their name and their current state. Next, coaching participants are asked to write their expectations for the weekend on little cards that are read aloud, gathered and clustered in an overview flipchart. This way, coaches can check if the contents agreed upon in an earlier skype meeting are still in focus. After presenting the timetable for the weekend, the actual coaching starts. At first, coaches implement some lineups on questions like “I know a lot about sustainability best practices from other universities” with the whole group in order to clarify present knowledge. This lineup also gives an opportunity to share knowledge in the group and ask other opinion-based questions. Afterwards, a visioning journey is undertaken and participants get the chance to share their visions, sometimes even forming a joint vision. The day ends with pizza delivery. On Saturday, a headline might be “Projects and Communication”. Starting with a brainstorming on possible projects at their university, the group soon reveals their underlying conflicts concerning informational hierarchies. Now, the coaches address this conflict and thereby lead over to a discussion on communication practice in group meetings. They use a headstand technique that asks participants about how the worst team meeting would look like and thus creates a more easygoing ambience. Next, the group infers characteristics of a good team meeting from it. All is captured on a nicely designed flipchart. This day is also accompanied by a number of energizer games, an input on best practices at other universities and an organizing platform for sustainability initiatives, a private journaling method, and skill development concerning project management tools. On Sunday, participants get to know the stakeholder-matrix as a method for strategic contact planning. Finally, they create a road map for their future projects, distribute responsibilities, give feedback to the coaches, and complete the weekend with a final energizer.

Table A3. Means (M), standard deviations (SD), Cronbach's Alpha, and correlations of transformed scales (and items, resp.) of pre-questionnaire participants. We reversed volunteer time and having a vision items so that their direction fitted transformed scales.

	M	*SD*	*α*/*r*	1	2	3	4	5	6	7	8	9	10	11	12	13	14
**1. Action skills**	4.19	1.17	.78														
**2. Having a vision**	4.79	1.47	-	.55													
**3. Group identification**	5.43	1.02	.78	.30	.31												
**4. Collaboration skills**	4.97	0.93	.56	.11	.14	.58											
**5. Efficacy affect**	5.48	1.03	.84	.23	.33	.51	.40										
**6. Efficacy**	5.31	0.87	.90	.38	.42	.37	.25	.47									
7. Self-efficacy	5.32	0.97	.79	.32	.35	.25	.13	.33	.86								
8. Collective efficacy	5.54	0.95	.87	.24	.27	.34	.34	.45	.86	.61							
9. Participative efficacy	4.93	1.25	.88	.41	.44	.33	.14	.38	.78	.51	.51						
**10. Behavior**	4.29	0.92	.72	.40	.41	.29	.07	.31	.35	.32	.21	.35					
11. Private	4.99	1.18	.73	.18	.23	.17	.12	.29	.30	.29	.27	.18	.66				
12. Indirect	5.71	1.14	.44	.28	.30	.27	.06	.32	.32	.35	.21	.24	.68	.44			
13. Protesting	2.64	1.39	.42	.24	.21	.09	.06	-.01	.10	.09	.00	.17	.62	.18	.19		
14. Volunteering	3.48	1.81	.38	.36	.33	.26	-.01	.23	.22	.16	.09	.33	.72	.15	.36	.34	
**15. Volunteer time**	4.83	3.66	-	.41	.21	.14	-.07	.09	.15	.04	.07	.29	.25	.09	.03	.15	.32

Table A4. Correlation table for one-item measures and main scales (non-transformed) of pre-questionnaire participants. Note that payment was erased from our dataset due to anonymity reasons.

	1	2	3	4	5	6	7	8	9	10	11	12	13	14	15	16	17	18
**1. Action skills**																		
**2. Having a vision**	.55																	
**3. Group ID**	.31	.32																
**4. Collab. skills**	.10	.14	.58															
**5. Efficacy affect**	.26	.34	.52	.39														
**6. Efficacy**	.38	.42	.39	.24	.50													
7. SE	.32	.36	.28	.13	.36	.87												
8. CE	.25	.27	.36	.33	.48	.87	.64											
9. PE	.41	.45	.34	.13	.40	.77	.52	.51										
**10. Behavior**	.40	.42	.30	.07	.33	.36	.33	.23	.36									
11. Private	.16	.23	.17	.13	.33	.32	.30	.30	.18	.67								
12. Indirect	.29	.31	.29	.05	.35	.36	.38	.26	.27	.69	.44							
13. Protesting	.24	.22	.08	.05	.00	.08	.07	.00	.16	.62	.19	.18						
14. Volunteering	.38	.35	.27	-.01	.22	.22	.16	.08	.34	.71	.13	.35	.36					
**15. Vol. time**	.40	.21	.14	-.07	.11	.13	.03	.06	.28	.25	.09	.03	.16	.33				
**16. Burnout**	.23	.15	-.08	-.25	-.17	-.02	-.06	-.14	.19	.12	-.05	.03	.07	.25	.41			
**17. Env. ID**	.25	.27	.18	.10	.28	.44	.44	.37	.28	.37	.44	.30	.15	.12	.08	.02		
**18. Stakeholder**	.57	.38	.25	.01	.28	.38	.32	.22	.44	.32	.14	.27	.17	.30	.34	.22	.16	
**19. Payment**	.21	.02	.05	.10	.02	.04	.00	.03	.06	-.03	.03	-.07	-.02	-.04	.43	.07	.03	.16

Note. SE=self-efficacy, CE=collective efficacy, PE=participative efficacy.

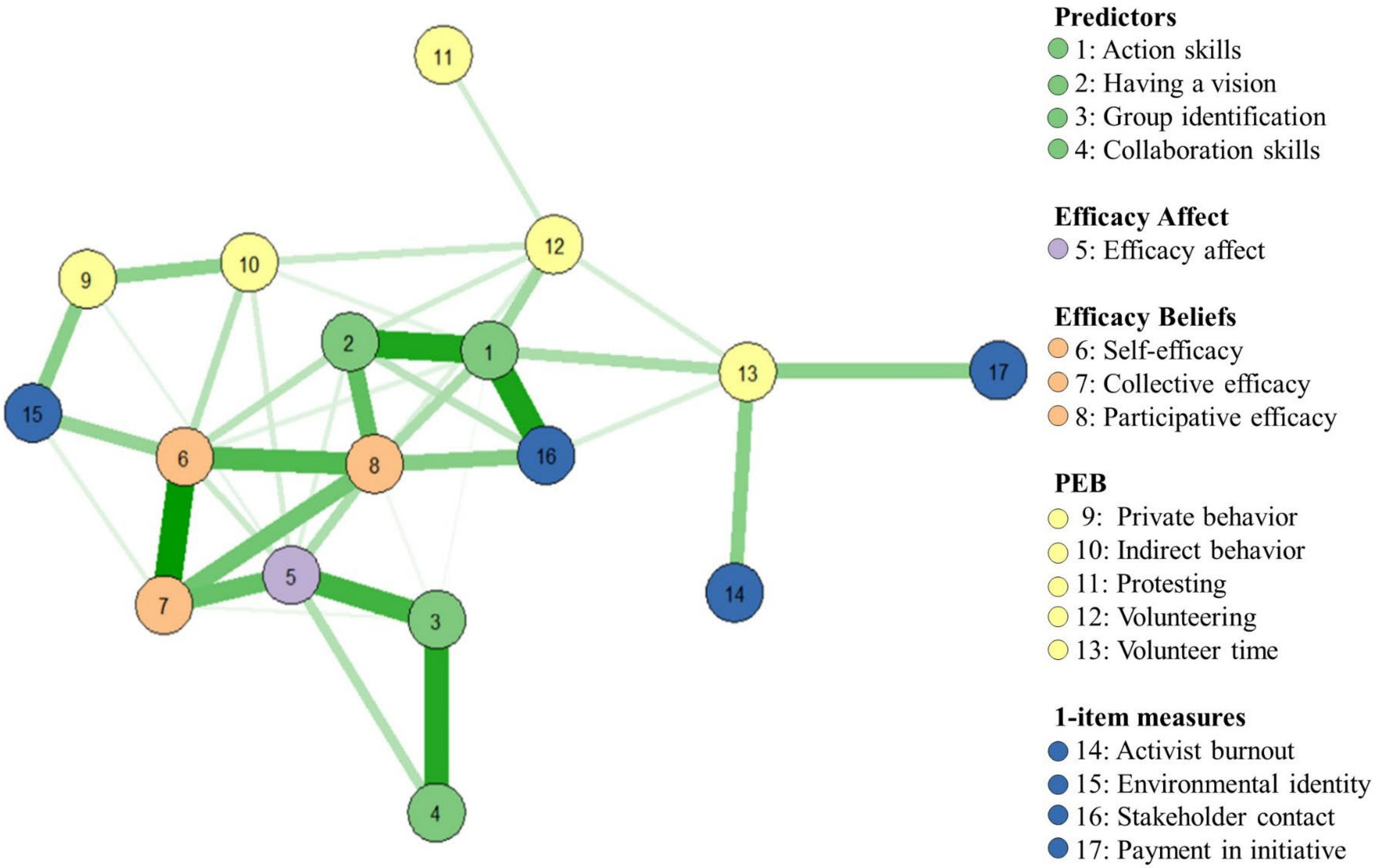

Figure A5. Correlation of one-item measures and main scales (non-transformed) visualized with Gaussian Plot. Note that subscales belonging to an overall scale share the same color and tend to form clusters. Bivariate correlations with an absolute value below 0.3 are not displayed for sake of clarity.

Figure A6. CFA of efficacy beliefs.

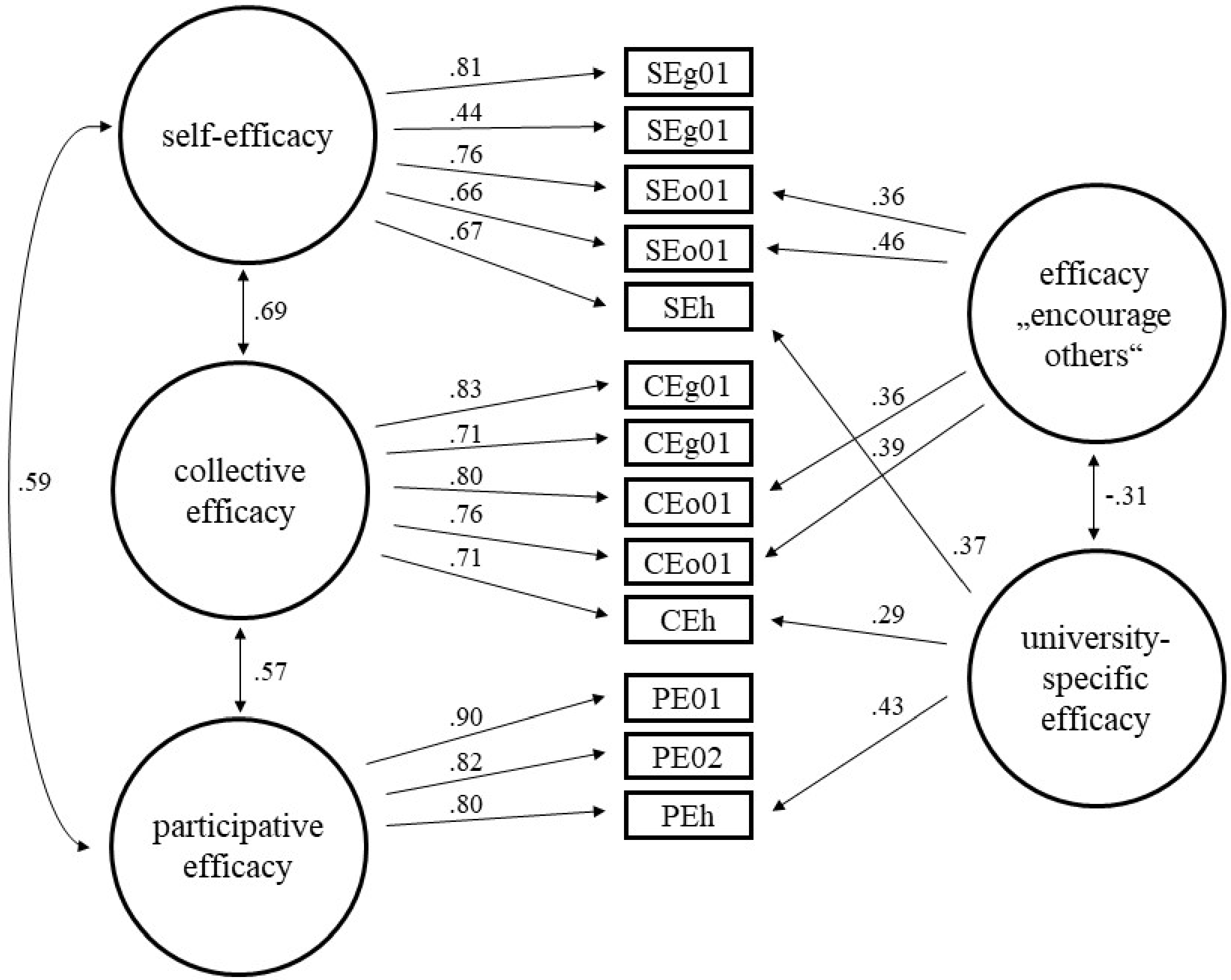

Figure A7. CFA of sustainability behavior.

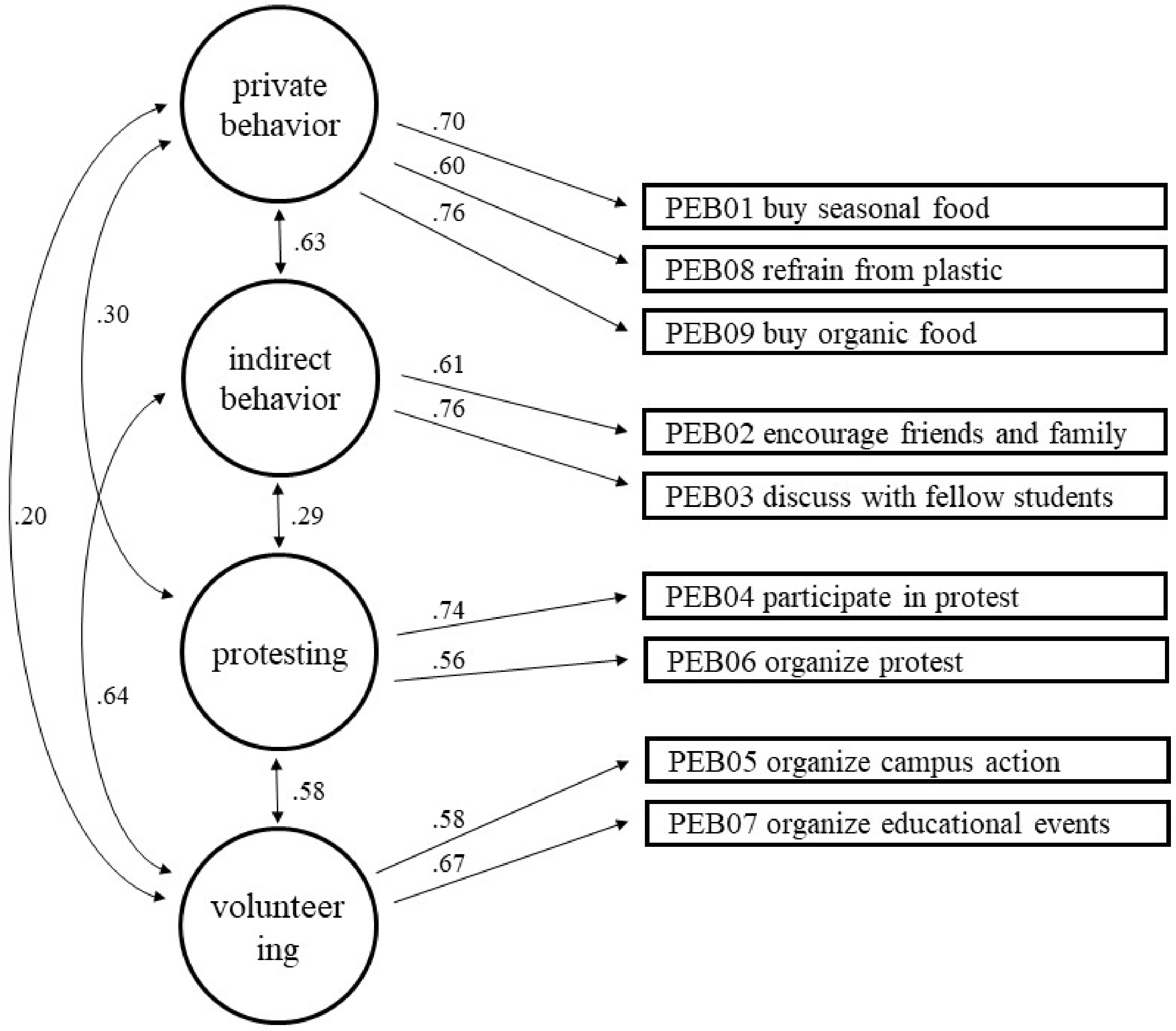

Table A8. Pre-post comparison with multilevel modeling. Square-transformed scales show similar results. Mean values are for complete sample of pre-post participants (n=165). Multilevel analyses contain only pre-post participants with university attribute.

			**multilevel modeling**			
	M(SD) pre	M(SD) post	*b* [95%-CI]	t	df	*p*
**Action skills**	4.17 (1.25)	5.39 (0.89)	1.14 [0.95, 1.34]	11.62	162	<.001
**Having a vision**	4.85 (1.49)	5.85 (1.00)	0.96 [0.71,1.21]	7.48	162	<.001
**Group identification**	5.48 (0.97)	5.91 (0.91)	0.38 [0.19,0.58]	3.91	163	<.001
**Collaboration skills**	5.01 (0.88)	5.63 (0.82)	0.63 [0.46,0.80]	7.38	163	<.001
**Efficacy affect**	5.57 (1.06)	5.91 (1.03)	0.30 [0.10, 0.51]	2.96	133	.004
**Efficacy**	5.33 (0.91)	5.65 (0.88)	0.28 [0.14, 0.42]	3.95	162	<.001
Self-efficacy	5.33 (0.99)	5.56 (0.97)	0.18 [0.03, 0.18]	2.34	162	.020
Collective efficacy	5.53 (1.00)	5.89 (0.93)	0.33 [0.16, 0.49]	3.88	162	<.001
Participative efficacy	5.00 (1.28)	5.41 (1.14)	0.38 [0.20, 0.57]	4.06	162	<.001
**Behavior**	4.45 (0.91)	4.53 (0.92)	0.06 [-0.06, 0.18]	1.05	150	.298
Private	5.14 (1.10)	5.08 (1.09)	-0.10 [-0.25, 0.05]	-1.29	150	.198
Indirect	5.88 (1.12)	5.81 (1.05)	-0.10 [-0.26, 0.06]	-1.22	161	.224
Protesting	2.63 (1.41)	2.82 (1.42)	0.21 [0.03, 0.38]	2.31	161	.022
Volunteering	3.79 (1.85)	4.09 (1.80)	0.30 [0.07, 0.53]	2.55	161	.012
**Volunteer time**	4.88 (3.36)	5.34 (3.69)	0.50 [0.08, 0.92]	2.35	161	.020
**Activist burnout**	2.64 (1.42)	2.84 (1.5)	0.20 [-0.05, 0.45]	1.55	162	.123
**Environmental ID**	5.45 (1.14)	5.65 (1.11)	0.17 [0.002, 0.34]	1.95	162	.053
**Stakeholder**	4.99 (1.70)	5.62 (1.26)	0.57 [0.30, 0.84]	4.22	133	<.001

Table A9. Pre-post comparison with paired t-tests. Square-transformed scales show similar results. Note that protesting, volunteering and volunteer time do not produce significant t-tests.

paired t-tests

	M(t1) -M(t0) [CI]	t	df	*r*	*p*
**Action skills**	1.22 [1.05, 1.39]	14.45	164	.748	<.001
**Having a vision**	0.99 [0.75, 1.24]	7.93	164	.527	<.001
**Group identification**	0.42 [0.24, 0.60]	4.68	164	.343	<.001
**Collaboration skills**	0.62 [0.46, 0.77]	7.93	164	.526	<.001
**Efficacy affect**	0.34 [0.15, 0.54]	3.46	135	.285	<.001
**Efficacy**	0.32 [0.16, 0.48]	4.05	164	.302	<.001
Self-efficacy	0.23 [0.06, 0.39]	2.72	164	.208	.007
Collective efficacy	0.36 [0.18, 0.54]	3.86	164	.288	<.001
Participative efficacy	0.41 [0.19, 0.63]	3.71	164	.279	<.001
**Behavior**	0.08 [-0.07, 0.23]	1.07	152	.087	.286
Private	-0.07 [-0.27, 0.14]	-0.64	152	.052	.523
Indirect	-0.07 [-0.27, 0.13]	-0.71	163	.055	.479
Protesting	0.19 [-0.01, 0.39]	1.89	163	.147	.060
Volunteering	0.30 [0.02, 0.62]	1.87	163	.145	.064
**Volunteer time**	0.45 [-0.12, 1.02]	1.56	163	.121	.121
**Activist burnout**	0.20 [-0.11, 0.51]	1.26	164	.098	.211
**Environmental ID**	0.21 [0.01, 0.41]	2.02	164	.156	.045
**Stakeholder**	0.63 [0.32, 0.94]	4.01	135	.326	<.001

Table A10. Descriptives, multilevel unstandardized regression coefficients, confidence intervals, and significance tests for participants who filled out pretest, posttest and follow-up. Scales represent shortened scales of follow-up questionnaire (items marked with * in questionnaire).

	M(SD) pre (n=34)	M(SD) post (n=33)	M(SD) FU (n=34)	*b* (t2-t0) [CI]	*p*
Group identification	5.38(1.12)	6.00(0.83)	5.90(1.06)	0.24[-0.02,0.50]	.065
Efficacy affect	5.85(0.76)	6.16(0.57)	5.73(0.98)	-0.05[-0.26, 0.16]	.632
Self-efficacy	5.75(1.14)	5.91(1.09)	5.72(1.12)	-0.02[-0.32,0.28]	.904
Collective efficacy	5.75(1.02)	6.08(0.74)	6.03(1.03)	0.13[-0.20, 0.46]	.431
**Participative efficacy**	**4.88(1.70)**	**5.55(1.15)**	**5.50(1.05)**	**0.31[0.006,0.61]**	**.046**
Private behavior	5.23(1.03)	5.31(0.78)	5.42(0.89)	0.11[-0-03,0.26]	.116
Indirect behavior	5.71(1.12)	5.89(0.92)	5.74(1.16)	0.01[-0.21,0.23]	.942
Protesting	2.83(1.46)	3.06(1.55)	2.97(1.23)	0.04[-0.17,0.24]	.709
**Volunteering**	**3.42(1.76)**	**3.86(1.66)**	**4.82(1.66)**	**0.71[0.38,1.04]**	**<.001**
Volunteer time	5.97(4.01)	6.94(4.97)	5.85(4.03)	0.09[-0.68,0.87]	.807

Note. If participants left their e-mail address at the end of our post-questionnaire, they were contacted with the invitation to a follow-up questionnaire about six months after their last participation and had the chance of winning one of three vouchers. Our follow-up questionnaire still included our total scale of sustainability behavior and efficacy affect. However, group identification was reduced to two items (r=.47), and actor-goal efficacy beliefs were reduced to a five-item-scale (α=.90) including two self-efficacy items (r=.62), two collective efficacy items (r=.67, p<.001 for all) and one participative efficacy item (particular items are marked in questionnaires S26/27). Since only n=35 filled out our follow-up questionnaire (22 females, 10 males; age M = 23.82 years, SD = 3.14), we mainly report descriptive results. All constructs except for sustainability behavior decreased in the follow-up questionnaire compared to the post questionnaire, however, participative efficacy and volunteering remained significantly stronger than in the pretest and some constructs show descriptive increases (e.g., group identification, collective efficacy, private behavior, protesting). However, we cannot cancel out that changes were due to longer group membership instead of the coaching weekend. Note that other than in the post test, follow-up participants did not differ significantly on all important scales from other pre-test participants (p>.05 for all).

Table A11. Fixed effect predictors of self-efficacy (pre-questionnaire).

	b	95% *CI*	*SE b*	pseudo R^2	*p*
(Intercept)	5.34	5.22, 5.44	.06		<.001
**Action skills**	**0.12**	**0.01, 0.23**	**.06**	**.013**	**.033**
**Having a vision**	**0.10**	**0.02, 0.19**	**.04**	**.017**	**.016**
**Group ID**	**0.19**	**0.04, 0.33**	**.07**	**.019**	**.011**
Collaboration skills	-0.01	-0.13, 0.16	.08	.003	.865

Overall pseudo R^2 =.120

Table A12. Fixed effect predictors of collective efficacy (pre-questionnaire).

	b	95% *CI*	*SE b*	pseudo R^2	*p*
(Intercept)	5.52	5.37, 5.65	.07		<.001
Action skills	0.05	-0.06, 0.15	.05	<.001	.361
Having a vision	0.06	-0.21,0.14	.04	.005	.132
**Group ID**	**0.18**	**0.05, 0.32**	**.07**	**.023**	**.009**
**Collaboration skills**	**0.19**	**0.05, 0.33**	**.07**	**.021**	**.001**

Overall pseudo R^2=.139

Table A13. Fixed effect predictors of participative efficacy (pre-questionnaire).

	b	95% *CI*	*SE b*	pseudo R^2	*p*
(Intercept)	4.95	4.80, 5.09	.07		<.001
**Action skills**	**0.16**	**0.03, 0.30**	**.07**	**.016**	**.021**
**Having a vision**	**0.24**	**0.13, 0.34**	**.05**	**.063**	**<.001**
**Group ID**	**0.34**	**0.17, 0.52**	**.09**	**.047**	**<.001**
Collaboration skills	-0.04	-0.22, 0.14	.09	<.001	.664

Overall pseudo R^2=.235

Table 14. Fixed effect predictors of sustainability behavior (pre-questionnaire participants).

	b	95% *CI*	*SE b*	pseudo R^2	*p*
(Intercept)	4.35	4.19, 4.52	.08		<.001
**Efficacy beliefs**	**0.17**	**0.03, 0.32**	**.08**	**.019**	**.022**
**Efficacy affect**	**0.18**	**0.06, 0.30**	**.06**	**.033**	**.003**
**Action skills**	**0.24**	**0.14, 0.34**	**.05**	**.080**	**<.001**
Having a vision	0.08	-.001, 0.16	.04	.011	.057
Group identification	0.01	-0.13, 0.15	.07	.004	.910
Collaboration skills	-0.12	-0.26, 0.02	.07	.009	.086
Overall pseudo R^2=.288					

Table A15. Fixed effect predictors of volunteer time (pre-questionnaire).

	b	95% *CI*	*SE b*	pseudo R^2	*p*
(Intercept)	4.76	4.11, 5.45	.33		<.001
Efficacy beliefs	-0.25	-0.84, 0.33	.30	<.001	.403
Efficacy affect	0.42	-0.05, 0.89	.24	.008	.086
**Action skills**	**0.80**	**0.40, 1.19**	**.20**	**.057**	**<.001**
Having a vision	0.10	-0.21, 0.41	.16	<.001	.534
**Group ID**	**0.68**	**0.12, 1.24**	**.29**	**.019**	**.019**
**Collaboration skills**	**-0.90**	**-1.44, -0.35**	**.28**	**.038**	**.002**
Overall pseudo R^2=.157					

Table A16. Fixed effect predictors of indirect behavior (pre-questionnaire).

	b	95% *CI*	*SE b*	pseudo R^2	*p*
(Intercept)	5.79	5.66, 5.94	.07		<.001
**Self-efficacy**	**0.29**	**0.12, 0.46**	**.09**	**.039**	**.001**
Collective efficacy	-0.01	-0.21, 0.17	.10	<.001	.846
Participative efficacy	-0.05	-0.18, 0.08	.07	<.001	.488
**Efficacy affect**	**0.25**	**0.09, 0.41**	**.08**	**.035**	**.002**
**Action skills**	**0.19**	**0.05, 0.32**	**.07**	**.026**	**.007**
Having a vision	0.03	-0.08, 0.13	.05	<.001	.644
Group identification	0.04	-0.15, 0.23	.10	<.001	.672
Collaboration skills	-0.11	-0.29, 0.08	.09	.001	.267

Overall pseudo R^2=.177

Table A17. Fixed effect predictors of protesting (pre-questionnaire).

	b	95% *CI*	*SE b*	pseudo R^2	*p*
(Intercept)	2.60	2.29, 2.84	0.14		<.001
Self-efficacy	0.07	-0.15, 0.29	0.11	<.001	.538
Collective efficacy	-0.09	-0.33, 0.15	0.12	<.001	.490
**Participative efficacy**	**0.25**	**0.08, 0.41**	**0.09**	**.030**	**.005**
Efficacy affect	-0.07	-0.27, 0.13	0.10	<.001	.524
**Action skills**	**0.29**	**0.12, 0.46**	**0.09**	**.042**	**.001**
Having a vision	0.07	-0.07, 0.20	0.07	<.001	.339
Group identification	-0.16	-0.39, 0.07	0.12	.003	.200
Collaboration skills	0.08	-0.16, 0.31	0.12	<.001	.532

Overall pseudo R^2=.124

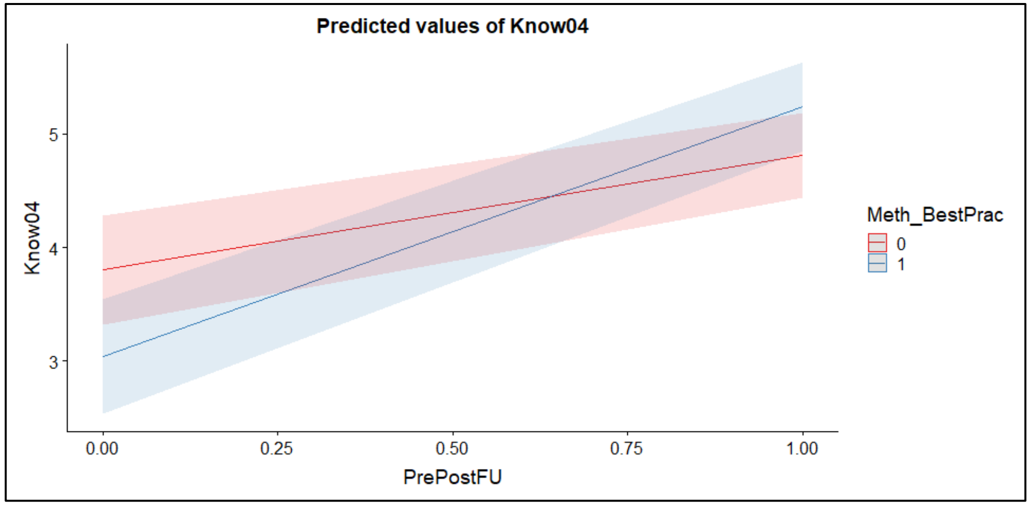

Figure A18. Interaction of method "Learning about best practices" and the item "I know best practices of other universities in the context of sustainable universities (e.g. Studium Oecologicum, etc.)".

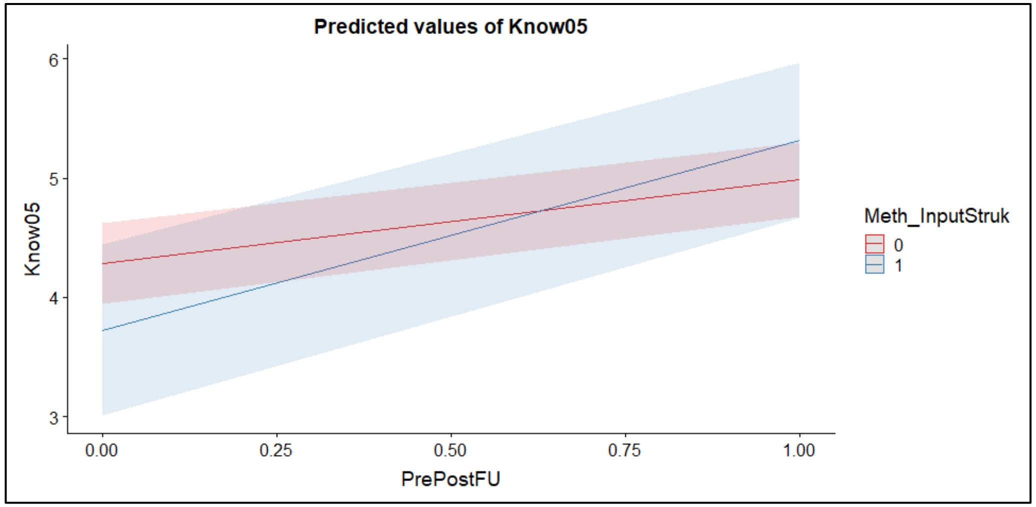

Figure A19. Interaction of method "Learning about university structures" and the item "I know structures of my university (e.g. function and composition of the AStA, role of the deanery, etc.)"

Table A20. Fixed effect predictors of activist burnout (pre-questionnaire participants).

	b	95% *CI*	*SE b*	pseudo R^2	*p*
(Intercept)	2.77	2.54, 3.01	0.12		<.001
**Self-efficacy**	**-0.38**	**-0.62, -0.13**	**0.13**	**.033**	**.004**
Collective efficacy	-0.27	-0.54, 0.001	0.14	.012	.055
**Participative efficacy**	**0.36**	**0.17, 0.54**	**0.10**	**.053**	**<.001**
Efficacy affect	-0.18	-0.40, 0.04	0.11	.007	.115
**Action skills**	**0.35**	**0.16, 0.54**	**0.10**	**.047**	**<.001**
Having a vision	0.03	-0.12, 0.18	0.08	<.001	.661
Group identification	0.03	-0.23, 0.30	0.14	<.001	.820
**Collaboration skills**	**-0.34**	**-0.60, -0.08**	**0.13**	**.022**	**.012**
Overall pseudo R^2=.192					

Exploratives A21.
Additional to our already reported analyses, we conducted a number of extra exploratory analyses so that other researchers can build their hypotheses with them:

A21.1. Efficacy affect as part of group identification and efficacy belief predictor
We controlled if group identification and efficacy affect could be empirically differentiated via factor analysis. EFA and CFA confirmed the differentiability of the two constructs (one-factor model: CFI=.766, AIC=9479; two-factor model: CFI=.913, AIC=9343). This alleviated our concerns that efficacy affect (concerning volunteer work) shared a dimension with group identification. Nevertheless, we left out positive affects towards the group in our operationalization of group identification. Therefore, it is indeed possible that efficacy affect exactly emphasizes this affective part of group identification. We were also curious if efficacy affect might serve as an efficacy predictor as proposed by Bandura (1997). As can be seen in table A22, efficacy affect was by far the most important predictor of efficacy believes (p<.001, pseudo R^2=.081), and all its subtypes (p<.05), and group identification even lost its predictiveness. Latent change models revealed that a change in efficacy affect is highly correlated with a change in self-efficacy (r=.72, p=.008), collective efficacy (r=.72, p=.005), and participative efficacy (r=.76, p=.001). Efficacy affect could be tested as a predictor of efficacy in future studies.

A21.2. Item-level associations

We created Gaussian plots in order to see relationships of main constructs with action skills and group identity at the item level (see figures A23/24). Concerning action skills, knowledge about best practices was related most strongly with sustainability behavior. Knowledge on sustainability at the university shows strong relations with having a vision which again relates to efficacy beliefs. Of all group identity variables, group belonging showed the strongest relations to sustainability behavior.

A21.3. Exploring method effects

Furthermore, we produced correlation tables of coaching methods and change scores for main scales. It turned out that efficacy beliefs were not affected by any specific method. However, action skills, group identification, efficacy affect, and protesting showed some significant relationships with various coaching methods (action skills: learning about sus. university, gallery walk, word café, systemic lineup, future workshop; group identification: journaling, systemic lineup; efficacy affect: competence figure, learning about sus. university, exchange on project ideas; protesting: energizer, wander molecules, case study, learning about university structure, learning about best practices, project-decision-matrix). Please inspect our data for further insights.

A21.4. Exploratory model tests

In order to integrate our findings into theories of collective action, we implemented several exploratory analyses. First, we built a model out of theoretical considerations drawn from the SIMCA model (van Zomeren et al., 2008), the EMSICA model (Thomas et al., 2011), Self-Efficacy Theory (Bandura, 1997), and Empowerment Theory (Zimmermann, 1990). Second, we had a look at Gaussian Graphs predicting our four types of sustainability behavior, this way gaining a better picture of correlative associations and possible mediation paths. Third, we repeated H3a analyses without action skills as predictor because we assume that specific action skills included in the scale (e.g., knowledge about university structures) might be a stronger result than precursor of volunteering. Furthermore, we removed negative predictors so that no positive predictor only became significant as a response to those negative predictors. Compared to the results of our third hypothesis, a slightly new picture arose in which having a vision played a more important role, and the effect of group identification was diminished because it mostly seemed to be a buffer for negative effects of collaboration skills on volunteering and volunteer time. Finally, we tested an exploratory path model and tried to find out which model fit the data best. A model fit the data best in which

- efficacy affect was predicted by group identification and having a vision,
- self-efficacy was predicted by efficacy affect and having a vision,
- participative efficacy was predicted by efficacy affect, having a vision and self-efficay,
- private behavior was predicted by efficacy affect,
- indirect behavior was predicted by efficacy affect and self-efficacy,
- protesting was predicted by having a vision, and

- volunteering was predicted by participative efficacy and having a vision (CFI=.969, RMSEA=.062, SRMR=.052).

Collective efficacy was not included in the over model because of a low fit, however, in a reduced model, it was positively predicted by efficacy affect, having a vision and collaboration skills (that was in turn predicted by group identification, CFI=.965, RMSEA=.081, SRMR=.036). In order to explore feedback loops, we conducted reverse multi-level and path analyses. Self-efficacy, collective efficacy and efficacy affect seem to be best predicted by private and indirect behavior. Participative efficacy, having a vision, action skills, and group identification were best predicted by volunteering and indirect behavior. Of course, those analyses cannot be interpreted causally and should be treated with caution due to their exploratory nature. All exploratory analyses can be found and reanalyzed with our script and an open dataset on OSF (Hamann et al., 2019).

Table A22. Fixed effect predictors of efficacy including efficacy affect (pre-questionnaire participants).

	b	95% *CI*	*SE b*	pseudo R^2	*p*
(Intercept)	5.33	5.21, 5.45	0.06		<.001
Action skills	0.07	-0.02, 0.16	0.05	.006	.130
**Having a vision**	**0.12**	**0.06, 0.19**	**0.03**	**.049**	**<.001**
Group identification	0.08	-0.04, 0.21	0.06	.003	.186
Collaboration skills	0.04	-0.08, 0.16	0.06	<.001	.506
**Efficacy affect**	**0.20**	**0.10, 0.30**	**0.05**	**.060**	**<.001**

Overall pseudo R^2=.255

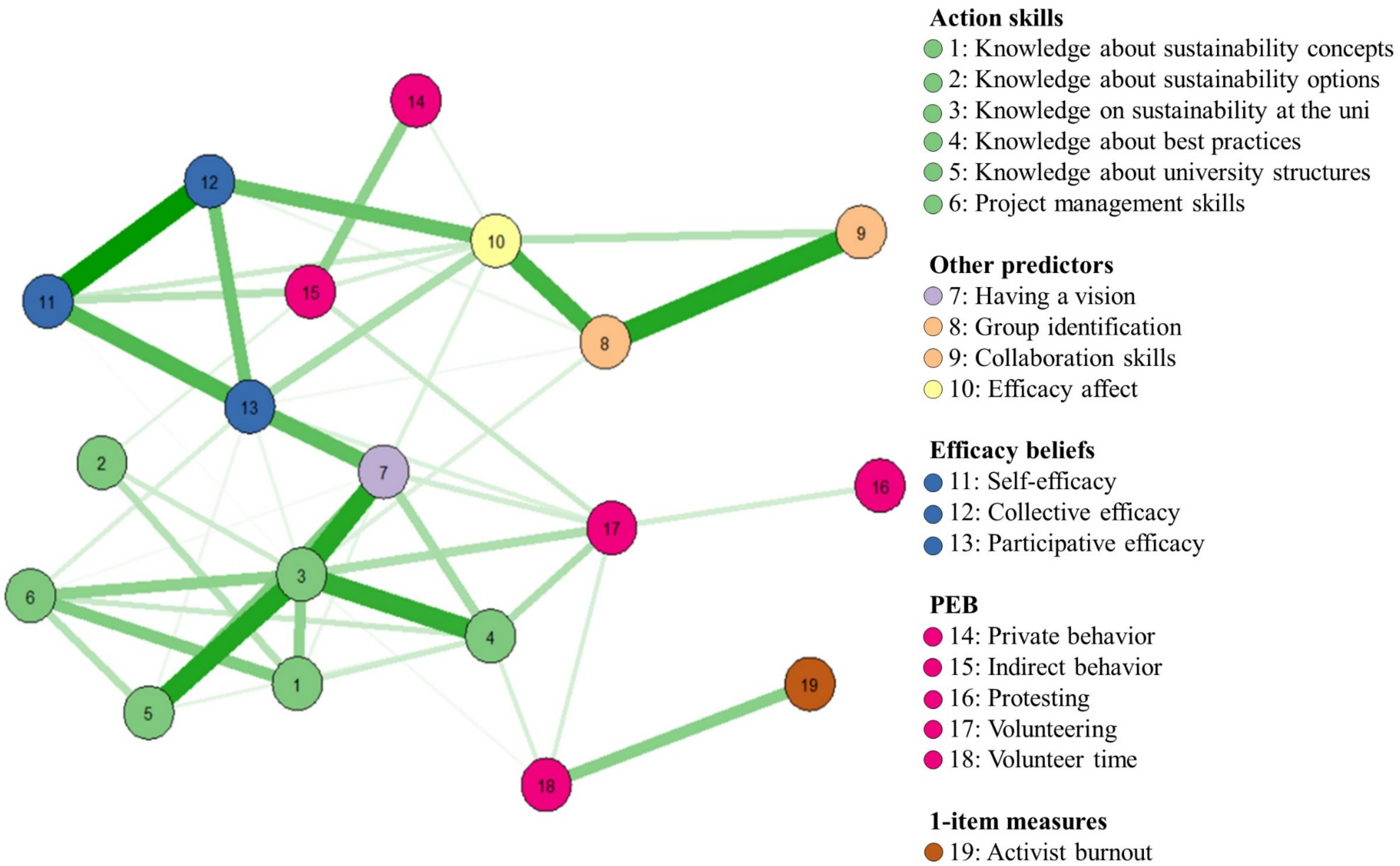

Figure A23. Gaussian Graph visualization of relationships of action skills items and burnout. Note that subscales belonging to an overall scale share the same color and tend to form clusters. Bivariate correlations with an absolute value below .30 are not displayed for sake of clarity.

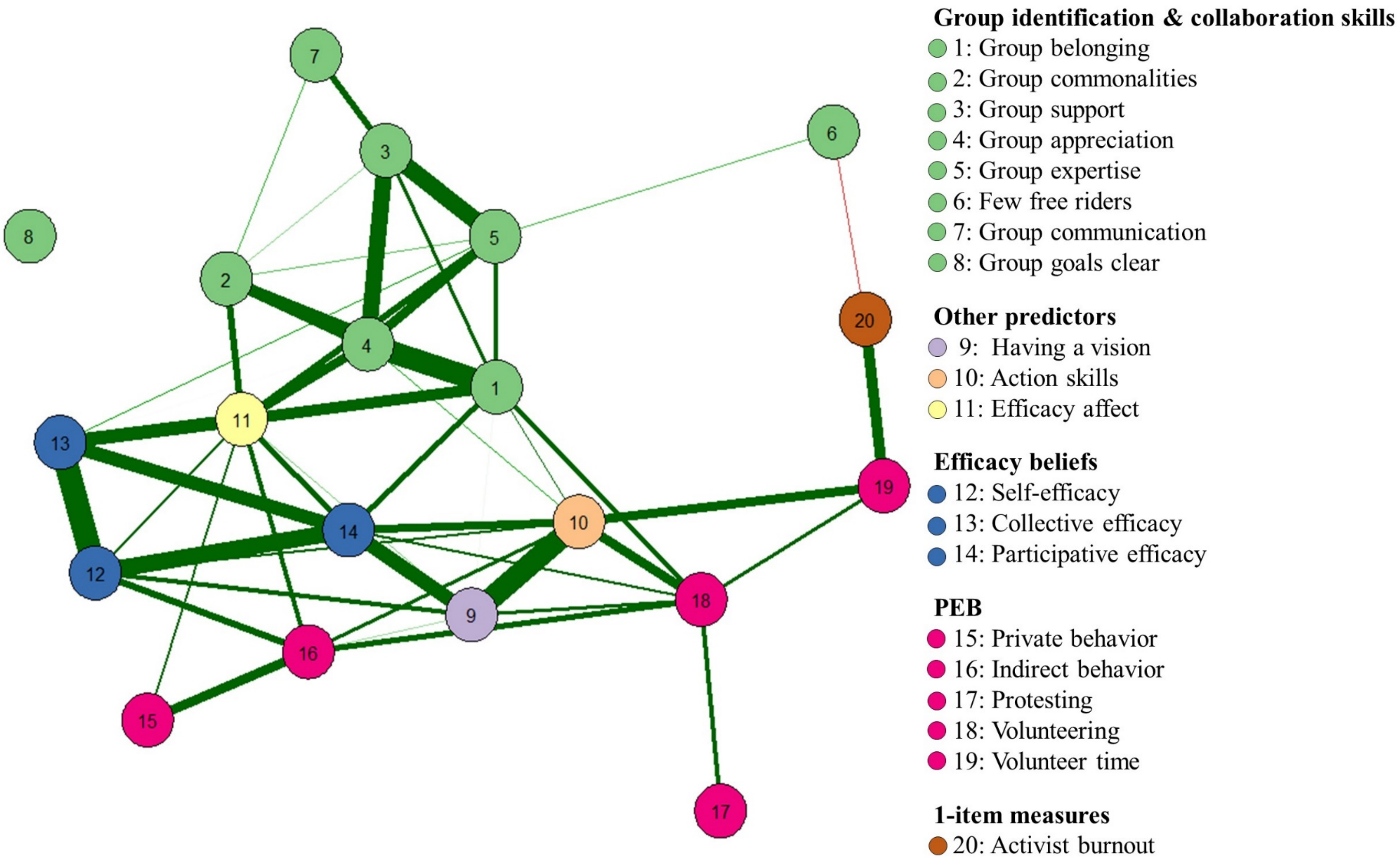

Figure A24. Gaussian Graph visualization of relationships of group identification items and burnout. Note that subscales belonging to an overall scale share the same color and tend to form clusters. Bivariate correlations with an absolute value below .30 are not displayed for sake of clarity.

English questionnaire A25.

(items marked with * were used in the follow-up questionnaire)

Personal Code

Following the Wandercoaching, there will once again be a short questionnaire. Please create a personalized code here, so that your future answers can be assigned to the answers from the current questionnaire. Your personalized code is composed of the first letter of your mother's first name, the first letter of your father's first name and the first letter of your birthplace. For example: Sarah, Bob, Hamburg gives the code "SBH".

1. Personalized Code (e.g., Sarah, Bob, Hamburg gives "SBH") *

Your personal knowledge

The following questions are all about your self-evaluation on certain topics and skills. The meaning of the numbers is shown above each question.1 stands for "I completely disagree" and 7 for "I completely agree". All other numbers are for grading your answers. There are no right or wrong answers. Please answer all questions as honestly and spontaneously as possible.

How much do you agree with the following statements?

- I am familiar with conceptual basics of sustainability (e.g. the triple bottom line model, strong vs. weak sustainability).
- I know about sustainable behavior alternatives. (e.g. for lowering the Co2- footprint).
- I am familiar with sustainability at the university (e.g. organizational structures, environmental management systems, etc.).
- I know best practices of other universities in the context of sustainable universities (e.g. Studium Oecologicum, etc.).
- I know structures of my university (e.g. function and composition of the AStA, role of the deanery, etc.).
- I know project management methods that I can use in my initiative work (for example Time Task Matrix, etc.).
- I have a vision of how a sustainable university could look like.
- I think of myself as an environmentally-friendly person.
- I feel able to contact stakeholders of my university (e.g. principal, cafeteria administration).

The initiative

The following questions are related to your sustainability initiative that participates in the Wandercoaching workshop. There are no right or wrong answers. Since your answers are anonymous, nobody can relate you to your answers. Please answers as honestly and spontaneously as possible.

How much do you agree with the following statements?

- I feel like I belong to the initiative. *
- I have a lot in common with other initiative members.
- I feel supported by other group members.
- The other group members make me feel appreciated.
- I rate other group members as competent.

- There are group members who rest on the work of others.
- I am satisfied with communication structures of our initiative.
- Our sustainability initiative has clear goals.
- I feel burned out because of my commitment.

In my work for the initiative I feel

- Hopeful*
- Motivated*
- Enthusiastic*

The effect

Here, you ‘re evaluating how much influence you, your sustainable initiative or you as a member of the sustainable initiative have. One question in each question block is related to the influence in a university context. Please mind this subdivision in the following three question blocks and answer every question honestly and spontaneously. Some questions may sound similar, nevertheless it is important for our evaluation that you answer all of them.

Here, it’s all about the influence of you as a person.

- I believe that I, through individual actions, can promote sustainable development. *
- I believe that I as an individual can achieve my goals in the field of sustainability.
- I believe that I as an individual can contribute so that others will behave more sustainably. *
- My sustainable action will encourage others to do the same.
- I believe that I, through individual actions, can promote sustainable university development.

This part is about the impact of your initiative.

- I believe that, through joint actions, we as an initiative can promote sustainable development. *
- I believe that we can achieve our common sustainability goals in our initiative.
- I believe that together, as an initiative, we can contribute so that others will behave more sustainably. *
- Our sustainable actions in the initiative will encourage others to do the same.
- I believe that, through joint actions, we as an initiative can promote sustainable university development.

This part is about your contribution in your initiative.

- I believe, that I as an individual can make a significant difference, so that we as an initiative can promote sustainable development. *
- I believe that I can make an important contribution, so that we as an initiative can achieve our common sustainability goals.
- I believe that I as an individual can make a significant difference, so that we as an initiative can promote sustainable university development.

Your Engagement

This block contains questions about your personal and group commitment for sustainability. This block is no longer about your personal evaluation, but about your actual actions. There are no right or wrong answers. Please answer all questions as honestly and spontaneously as possible.
How much do you agree with the following statements?

- I mainly buy seasonal food. *
- I try to convince my friends and family members of the importance of sustainable development. *
- I discuss with fellow students about sustainable development. *
- I participate in protests (demonstrations, rallies, occupations, etc.) that promote sustainable development. *
- I organize actions on the campus (info tables, flash mobs, etc.) *
- I organize protests (demonstrations, rallies, occupations, etc.) that promote sustainable development. *
- I organize educational events about sustainability topics. *
- I refrain from plastic (packaging). *
- I mainly buy organic products (e.g. food, washing agents, clothes). *

How many hours a week are you working or volunteering for the initiative? *
Name of your university:
In which semester do you study?
How old are you?
Gender (female/ male/ not specified)

Are you getting paid for your work in the initiative?

- Yes, I get paid for more than 6 hours a week
- Yes, I get paid for less than 6 hours a week
- No

Have you already participated in a Wandercoaching in the past?
My wishes for the Wandercoaching:
What I would like to say:

Questions only included in the post-questionnaire
At first, we would like to ask you for your evaluation of the wandercoaching workshop, the warm-up phase and platform n.

- I participated in the wandercoaching weekend.
- I participated in the warm-up phase
- I’m satisfied with the wandercoaching weekend.

- The coaches met our needs.
- The warm-up phase was a good preperation for the coaching weekend.
- I'm satisfied with the warm-up phase.
- I can orient myself at the platform n.
- I will use platform n in the future.
- What I liked best about the wandercoaching:
- What the wandercoaching changed for me/ my initiative:
- What I would do different in the wandercoaching/ what I'd wish for:
- Which support would you like to have for your future activities from the wandercoaches or from network n?

German original questionnaire A26.
(items marked with * were used in the follow-up questionnaire)
Im Anschluss an das Wandercoaching wird es erneut einen Kurzfragebogen geben. Damit Deine zukünftigen Antworten den Antworten aus der jetzigen Befragung zugeordnet werden können, erstelle hier bitte einen personalisierten Code. Dieser setzt sich zusammen aus dem ersten Buchstaben des Vornamens deiner Mutter, dem ersten Buchstaben des Vornamens deines Vaters und dem ersten Buchstaben deines Geburtsortes. Zum Beispiel ergibt Sabine, Bernd, Hannover den Code "SBH".
Personalisierter Code (z.B. Sabine, Bernd, Hannover ergibt "SBH") *

Deine persönlichen Kenntnisse
Im Folgenden geht es um Deine Selbsteinschätzung zu gewissen Themenbereichen und Fähigkeiten. Die Bedeutung der Zahlen ist jeweils angegeben, wobei 1 für „stimme überhaupt nicht zu" steht und 7 für „stimme voll und ganz zu". Die anderen Zahlen dienen der Abstufung deiner Antworten. Es gibt keine richtigen oder falschen Antworten. Bitte beantworte alle Fragen möglichst ehrlich und spontan.
- Ich kenne mich mit den konzeptionellen Grundlagen der Nachhaltigkeit aus (z.B. das Drei-Säulen-Modell, starke vs. schwache Nachhaltigkeit).
- Ich kenne mich bei nachhaltigen Verhaltensalternativen aus (z.B. um den CO2-Fußabdruck zu verringern).
- Ich kenne mich mit Nachhaltigkeit an der Hochschule aus (z.B. Organisationsstrukturen, Umweltmanagementsysteme, etc.).
- Ich kenne Beispiele des Gelingens von anderen Universitäten aus dem Kontext nachhaltiger Hochschule (z.B. Studium Oecologicum, etc.).
- Ich habe eine Vision, wie eine Hochschule in nachhaltiger Entwicklung aussehen könnte.
- Ich kenne die Hochschulstrukturen meiner Universität (z.B. Funktion und Zusammensetzung vom AStA, Rolle des Dekanats, etc.).
- Ich kenne Projektmanagement-Methoden, die ich in meiner Initiativen-Arbeit nutzen kann (z.B. Zeit-Aufgaben-Matrix, etc.).
- Ich denke von mir selbst, dass ich eine umweltschützende Person bin.

Die Initiative

Die folgenden Fragen beziehen sich auf Deine Nachhaltigkeits-Initiative, mit der Du am Wandercoaching teilnimmst. Es gibt keine richtigen oder falschen Antworten und durch die vollkommene Anonymisierung wird niemand Deine Antworten zuordnen können. Bitte beantworte alle Fragen möglichst ehrlich und spontan.

- Ich fühle mich der Initiative zugehörig. *
- Ich habe viel mit anderen Menschen in der Initiative gemeinsam. *
- Ich fühle mich von den anderen Gruppenmitgliedern unterstützt.
- Ich fühle mich von den anderen Gruppenmitgliedern wertgeschätzt.
- Ich schätze die anderen Gruppenmitglieder als kompetent ein.
- Es gibt Gruppenmitglieder, die sich auf der Arbeit anderer ausruhen.
- Unsere Nachhaltigkeitsinitiative hat klare Ziele.
- Ich bin mit den Kommunikationsstrukturen in unserer Initiative zufrieden.
- Ich fühle mich durch mein Engagement ausgebrannt.

In meinem Engagement für die Initiative fühle ich mich…

- hoffnungsvoll
- motiviert
- enthusiastisch

Deine Wirkung

Hier gibst Du Deine Einschätzung ab, welchen Einfluss Du selbst, Deine Nachhaltigkeits-Initiative bzw. Du als Mitglied der Nachhaltigkeits-Initiative hast. Bitte achte auf diese Unterteilung in den folgenden drei Frageblöcken und beantworte alle Fragen möglichst ehrlich und spontan.

Hier geht es um den Einfluss von Dir als Person.

- Ich glaube, dass ich durch eigenes Handeln nachhaltige Entwicklung vorantreiben kann. *
- Ich glaube, dass ich als Individuum meine Ziele im Bereich der Nachhaltigkeit erreichen kann.
- Ich denke, dass ich als Individuum dazu beitragen kann, dass auch andere Menschen sich nachhaltiger verhalten. *
- Mein nachhaltiges Handeln wird andere motivieren, dasselbe zu tun.
- Ich glaube, dass ich durch eigenes Handeln nachhaltige Hochschulentwicklung vorantreiben kann.

Hier geht es um den Einfluss Deiner Initiative.

- Ich glaube, dass wir als Initiative durch gemeinsames Handeln nachhaltige Entwicklung vorantreiben können. *
- Ich glaube, dass wir in der Initiative die gemeinsamen Nachhaltigkeitsziele erreichen können.
- Ich denke, dass wir als Initiative gemeinsam dazu beitragen können, dass auch andere Menschen sich nachhaltiger verhalten. *

- Unser nachhaltiges Handeln in der Initiative wird andere motivieren, dasselbe zu tun.
- Ich glaube, dass wir als Initiative durch gemeinsames Handeln nachhaltige Hochschulentwicklung vorantreiben können.

Hier geht es um den Beitrag von Dir in Deiner Initiative.

- Ich glaube, dass ich als Individuum einen signifikanten Unterschied machen kann, sodass wir als Initiative nachhaltige Entwicklung vorantreiben können. *
- Ich glaube, dass ich einen wichtigen Beitrag leisten kann, sodass wir in der Initiative unsere gemeinsamen Nachhaltigkeitsziele erreichen können.
- Ich glaube, dass ich als Individuum einen signifikanten Unterschied machen kann, sodass wir als Initiative nachhaltige Hochschulentwicklung vorantreiben können.

Dein Engagement

Dieser Block beinhaltet Fragen zu Deinem persönlichen und gruppenbezogenen Engagement für Nachhaltigkeit. Es geht nun nicht mehr um Deine Einschätzung zu Themen, sondern um Dein tatsächliches Handeln. Dabei gibt es keine richtigen oder falschen Antworten. Bitte beantworte alle Fragen möglichst ehrlich und spontan. [1: trifft überhaupt nicht zu – 7 trifft voll und ganz zu]

- Ich kaufe hauptsächlich saisonale Nahrungsmittel. *
- Ich versuche, Familienmitglieder und Freund_innen zu überzeugen, dass nachhaltige Entwicklung wichtig ist. *
- Ich diskutiere mit Kommiliton_innen über nachhaltige Entwicklung. *
- Ich nehme an Protesten (Demonstrationen, Kundgebungen, Besetzungen, etc.) teil, die sich für nachhaltige Entwicklung einsetzen. *
- Ich organisiere Petitionen zum Thema nachhaltige Entwicklung. *
- Ich organisiere Proteste (Demonstrationen, Kundgebungen, Besetzungen, etc.), die sich für nachhaltige Entwicklung einsetzen. *
- Ich organisiere Bildungsveranstaltungen zu Nachhaltigkeitsthemen. *
- Ich kaufe überwiegend Bioprodukte (z.B. Nahrung, Waschmittel, Kleidung). *
- Ich verzichte vorwiegend auf Plastik(-verpackungen). *

Wie viele Stunden pro Woche engagierst Du Dich für die Initiative? [0-20] *
Name Deiner Hochschule:
In welchem Semester studierst Du?
Wie alt bist Du?
Geschlecht: männlich, weiblich, anderes
Wirst Du für Deine Arbeit in der Initiative bezahlt?

- Ja, ich werde für mehr als 6 Stunden pro Woche bezahlt
- Ja, ich werde für weniger als 6 Stunden pro Woche bezahlt

- Nein

Hast Du in der Vergangenheit bereits an einem Wandercoaching teilgenommen? [ja/nein]
Das erhoffe ich mir vom Wandercoaching:
Was ich noch sagen möchte:

German questions only included in the post-questionnaire

Als erstes möchten wir Dich um Deine Einschätzungen zum Wandercoaching-Workshop, zur Aufwärmphase und zur plattform n bitten.

- Ich habe am Wandercoaching-Wochenende teilgenommen.
- Ich habe an dem Aufwärmworkshop aktiv teilgenommen.
- Ich bin zufrieden mit dem Wandercoaching-Wochenende. [Zustimmung sehr-nicht]
- Die Wandercoaches sind gut auf unsere Bedürfnisse eingegangen. [Zustimmung sehr-nicht]
- Der Aufwärmworkshop war eine gute Vorbereitung auf das Coaching-Wochenende.
- Ich bin zufrieden mit dem Aufwärmworkshop.
- Ich finde mich auf der plattform n zurecht.
- Ich werde die plattform n zukünftig nutzen.
- Das hat mir beim Wandercoaching am besten gefallen:
- Das hat das Wandercoaching bei mir/ in unserer Initiative verändert:
- Das würde ich beim Wandercoaching anders machen/das hätte ich mir beim Wandercoaching noch gewünscht:
- Welche Begleitung und Unterstützung wünschst Du Dir im weiteren Verlauf und bei den zukünftig anstehenden Aktivitäten von den Wandercoaches bzw. vom netzwerk n?
